# Alleviation of Salinity Stress in Peanut by Application of Endophytic Bacteria

**DOI:** 10.3389/fmicb.2021.650771

**Published:** 2021-04-14

**Authors:** Kamal K. Pal, Rinku Dey, Dharmesh N. Sherathia, Devidayal, Shamsudheen Mangalassery, Arvind Kumar, Rupal B. Rupapara, Mona Mandaliya, Priya Rawal, Roshani A. Bhadania, Manesh Thomas, Mili B. Patel, Priyanka Maida, Bhagwat D. Nawade, Suhail Ahmad, Pitabas Dash, T. Radhakrishnan

**Affiliations:** ^1^Section of Microbiology, ICAR-Directorate of Groundnut Research, Junagadh, India; ^2^ICAR-Central Arid Zone Research Institute, Kukma, India; ^3^Division of Crop Improvement, ICAR-Central Soil Salinity Research Institute, Karnal, India

**Keywords:** salinity, endophyte, alleviation, peanut, growth-promotion, ROS scavenging

## Abstract

The development of salinity affects 7% of the world’s land surface, acting as a major constraint to crop productivity. This study attempted to use the co-evolving endophytes of peanut to alleviate salinity stress and enhance the yield of peanut. Diverse and different tissue colonizing endophytes were isolated from peanut and screened *in vitro* by seed germination bioassay imposing gradients of salinity, with two cultivars TG37A (susceptible) and GG2 (moderately resistant), in potted conditions using saline irrigation water. Finally, nine endophytes capable of producing IAA and ACC-deaminase, promoting root growth and yield in potted conditions were selected for further evaluation in field conditions. They were evaluated with saline water (1.5–2.0 dS/m) in saline soil with susceptible cultivar TG37A. Simultaneously, three endophytes (*Bacillus firmus* J22N; *Bacillus tequilensis* SEN15N; and *Bacillus* sp. REN51N) were evaluated with two cultivars, GG2 and TG37A, during rainy and post-rainy seasons with elevated salinity. The application of endophytes like *Bacillus firmus* J22N and *Bacillus* sp. REN51N enhanced the pod and haulm yield of peanuts by 14–19% across cultivars, salinity, and seasons. In addition, there was significant modulation in parameters like relative water content; production of enzymes like superoxide dismutase (SOD), glutathione reductase (GR), catalase (CAT), ascorbate peroxidase (APX), lipid peroxidase (POD), and H_2_O_2_ content in leaf; and uptake of potassium. The activities of the enzymes involved in scavenging reactive oxygen species (ROS) increased with salinity, and further increased with endophytes like *Bacillus firmus* J22N, *Bacillus tequilensis* SEN15N, and *Bacillus* sp. REN51N. There was an enhanced accumulation of proline, reduced level of phenol and H_2_O_2_, and enhanced uptake of potassium with the inoculation of endophytes. This improved scavenging capacity of plants by endophytic modulation of ROS scavengers, uptake of K, production of ACC deaminase and IAA, root and biomass growth, modulation in relative water content, and enhanced accumulation of osmoprotectant might be the reasons of alleviation of salinity stress. Endophytes could have alleviated salinity stress in peanuts, indicating the mechanisms and potential of peanuts at the field level. These endophytes could be applied to bring agricultural sustainability to salinity-affected areas in the future. Furthermore, few genera viz. *Kocuria*, *Brevundimonas*, *Agrococcus*, *Dietzia*, and *Kytococcus* were observed in peanut tissue for the first time.

## Introduction

Salinity affects about 20% of all irrigated agricultural fields and over 7% of the world’s land surface (Szabolcs, 1994). It is estimated that approximately 50% of arable land will be affected by salinity stress by the year 2050 (Munns and Tester, 2008; Shrivastava and Kumar, 2015). This is likely to impact the global food production needed to feed over 9.6 billion people, which the world population is estimated to reach by 2050. Increasing concentrations of salt in soil and or irrigation water is a major threat to agricultural production in arid and semi-arid regions. It is anticipated that because of the build-up of salinity in soil, there will be a drastic reduction in crop yield by inhibition of seed germination, seedling growth, flowering, and fruit set (Sairam and Tyagi, 2004). Almost all physiological processes like respiration, photosynthesis, nitrogen fixation and other metabolic and enzymatic processes are affected by soil salinity, resulting in stunted growth and a decrease in farm productivity (Shabala and Munns, 2012; Acosta-Motos et al., 2017; Shabala, 2017). It also disrupts the cellular osmotic balance (Krasensky and Jonak, 2012), and increases oxidative stresses by generating reactive oxygen species (ROS). Soil salinity, usually NaCl, may also reduce plant growth by ion toxicity and water deficits (Wu et al., 2013). Crop losses are predicted to reach approximately US$27.3 billion annually (Qadir et al., 2014). In the changing climate scenario, the impact of salinization is likely to increase further, necessitating special efforts to maintain sustainable crop yield under salt stress (Turral et al., 2011; Suarez et al., 2015).

Generally, stressed plants induce the production and accumulation of osmolytes, i.e., proline and glycine betaine (Qureshi et al., 2013; Alam et al., 2019) in response to salinity stress. Stressed plants are also reported to induce production of non-enzymatic, i.e., phenolics, flavonoids, and glutathione (Alam et al., 2019); and enzymatic antioxidants, i.e., peroxidase, catalase, as well as the enzymes involved in the ascorbate–glutathione cycle (Khaliq et al., 2015; Talbi et al., 2015; Khan et al., 2020) to mitigate the oxidative damage during salinity stress (Zhu, 2002). Plants growing in saline soil frequently increase their ethylene production to initiate programmed cell death (apoptosis) (Trobacher, 2009), which in turn leads to tissue senescence and stunted growth. Thus, a reduction in ethylene production inside plants could act as a growth stimulator under stress. Although breeding of tolerant crop varieties is an option, to date there has been limited success in developing such salt-tolerant crop plants (Schubert et al., 2009), necessitated alternate ameliorating or management strategies to achieve sustainable crop growth in soils affected by salinity. The modification in root growth, enhanced uptake of K^+^ concomitantly with a reduction in Na^+^ intake, and up or downregulation of other metabolic processes (Compant et al., 2005; Ryan et al., 2008; Khaliq et al., 2015; Ali et al., 2017; Li et al., 2019; Fan et al., 2020) are also responsible for imparting salinity tolerance. Application of epigallocatechin-3-gallate, salicylic acid, kinetin, silicon, aspirin, Ag-nanoparticals, and spermidine externally have also been found to alleviate abiotic stresses in many plants (Ahammed et al., 2018; Ahanger et al., 2018, 2020; Hussain et al., 2018; Kaur et al., 2018; Zhang et al., 2018; Alam et al., 2019; Ahmad et al., 2019; Khan et al., 2020).

Alternate management strategies include the use of beneficial microbes (endophytes, PGPR, etc.) capable of producing IAA for plant growth and ACC-deaminase (Glick et al., 2007; Raghuwanshi and Prasad, 2018; Saikia et al., 2018) for reducing ethylene level in stress; and exo-polysaccharides (Sun et al., 2020) for chelating Na^+^. Such microbes are also capable of reducing salt stress in plants through the production of ROS scavenging enzymes, modulating osmotic adjustment, enhancing uptake of K to counteract Na, and modulation of signaling pathways, etc. (Sairam and Tyagi, 2004; Compant et al., 2005; Ryan et al., 2008; Fan et al., 2020). Each of the nearly 300000 plant species harbor one or more endophytes that helps them to circumvent many abiotic and biotic stresses (Strobel and Daisy, 2003; Brundrett, 2006). Inoculation of *Pseudomonas pseudoalcaligenes* in rice alleviated salinity stress (Jha et al., 2011); *Klebsiella pneumoniae* in wheat fixed nitrogen (Iniguez et al., 2004); and *Enterobacter sakazaki* in soybean enhanced plant growth (Kuklinsky-Sobral et al., 2004). In peanut, the colonization of *Enterobacter* spp. and *Klebsiella* spp. inside the root nodules of peanut enhanced yield (Gimenez-Ibanez et al., 2009). Similarly, inoculation of endophytic *Bacillus* sp., *Paenibacillus* sp., and *Pantoea dispersa* obtained from peanut seeds modulated the metabolism of the peanut to modulate growth (Li et al., 2019; Fan et al., 2020). However, very little is known about the role of the endophytic modulation of physiological and biochemical parameters leading to the alleviation of salinity stress in peanuts.

Peanut (*Arachis hypogaea* L.) is a major oilseed crop in India. However, the crop is susceptible to salinity, and productivity is severely affected (Meena et al., 2012). As peanut cultivars tolerant to salinity are not available, we hypothesized that the use of co-evolving endophytic bacteria could provide succor to peanut against salinity stress, and impact plant growth and yield. In the present study, endophytic bacteria, isolated from different tissues of peanut, were evaluated for their role in alleviating salinity stress, along with plant growth promotion and yield enhancement of field conditions. The possible mechanisms of alleviation of the salinity stress of peanut by endophytes were also investigated.

## Materials and Methods

### Isolation of Endophytic Bacteria

The endophytic bacteria were isolated from the internal tissues of the root, stem, and seed of 80-day old peanut plants (cultivar GG2), grown in farmers’ fields affected by salinity (EC 4.5 dSm^–1^) in Kutch, Gujarat, India. Three samples of healthy plants, each from three different geographical locations between N 23°12.806′ and E 069°47.576′ to N 23°19.690′ and E 070°08.911′, were collected during the rainy and post-rainy seasons of 2010 and 2011 for the isolation. Similarly, endophytes were also isolated from peanut (cultivar TG37A) grown in moisture-deficit stress conditions (experimental plots; three samples of three plants each from three different locations; 70-day old plants) at ICAR-Directorate of Groundnut Research (21°31′N, 70°36′E), Junagadh, Gujarat, India following Kishore et al. (2005) with modifications. Peanut plants (cultivar GG2) collected from salinity affected farmers’ fields in the Kutch district of Gujarat (India) were brought to the laboratory at Junagadh, Gujarat by keeping the samples in polythene bags kept in insulated boxes during the summer season of 2010.

The temperatures inside the boxes were maintained at –20°C using gel packs, to prevent the samples spoiling. Fresh samples, obtained from the moisture-deficit stress conditions (no irrigation water after emergence, until harvest) of experimental plots in Junagadh, Gujarat, India were processed separately. For isolation of endophytic bacteria, 5 g of cleaned stem/root (cut into 3–5 cm pieces), were surface sterilized with 4% sodium hypochlorite solution for 3 min followed by 90% alcohol for a further 3 min, and finally with 70% ethanol for 3 min. Traces of alcohol were removed by rinsing the samples in sterile water five times.

The samples were then rolled onto Luria Bertani Agar (M1151, HiMedia) and Tryptone Soya Agar (TSA) (M290, HiMedia) plates to check surface contamination. They were then homogenized in 25 ml of sterilized phosphate buffer using a sterile mortar and pestle. Appropriate dilutions of these suspensions were plated onto Luria Bertani (LB) and TSA agar amended with 100 μg/ml cycloheximide to prevent fungal growth and incubated for 72 h at 28°C.

From each sample, single colonies of predominant strains with distinct morphologies and well separated from the others were picked up, sub-cultured, and preserved as glycerol stocks at –70°C. For isolation of the seed endophytes, 5 g of seeds of GG2 and TG37A, grown in the above-mentioned conditions, were directly placed into a sterile beaker and surface sterilized with 0.1% HgCl_2_ for 5 min. Thereafter, the protocol followed for the processing of root and stem samples was employed. The aliquot was transferred to 90 ml water blank and serially diluted, followed by spreading 100 μl on agar media plates and incubated at 28°C for 72 h.

### Characterization of Endophytes for Salinity Tolerance

To determine the inherent level of tolerance of the endophytes to salinity, the purified and morphologically distinct endophytes were grown in liquid Luria broth at 28°C for 24 h. An aliquot (5 μl) was spotted onto Luria Agar in triplicates, amended with 0% (control), 2.5, 5, 7.5, 10, 12.5, and 15% NaCl and incubated at 28°C for 72 h. Observations were recorded at 24 h intervals.

### Characterization of Endophytes for ACC Deaminase Activity and IAA Production

The presence of IAA-like substances was detected and quantified following the method of Sarwar and Kremer (1995) in L-tryptophan agar. One ml each of 24 h growth of the isolates in Kings’ B (King et al., 1954) broth was pour plated into L-tryptophan agar in triplicate and incubated at 28°C for 24 h in the dark. After incubation, the agar growth beads (three beads, approximately 0.24 cm^3^) were placed in freshly prepared Salkowsky reagent (Sarwar and Kremer, 1995) in triplicate, from each Petri dish and incubated in the dark for 30 min for the development of pink color, when they were measured spectrophotometrically at 595 ηm, using IAA as standard. The amount of IAA produced was expressed as μg/ml. The capacity of the newly isolated strains to produce ACC deaminase was determined as described (Glick et al., 1995). The endophytic isolates were screened for ACC deaminase activity on the sterile minimal DF (Dworkin and Foster) salts media amended with 3 mM ACC (1-cyclopropoane-1-carboxylic acid) as the sole nitrogen source (Dworkin and Foster, 1958; Penrose and Glick, 2003).

The inoculated plates were incubated at 28°C for 3 days, and growth was monitored on a daily basis. The colonies growing on the plates were taken as ACC deaminase producers. The quantitative assessment of ACC deaminase activity of ACC-deaminase positive endophytes was done spectro-photometrically in terms of the production of α-ketobutyrate from ACC at 540 ηm by comparing with the standard curve of α-keto-butyrate (Honma and Shimomura, 1978). The protein was estimated by Bradford reagent (Bradford, 1976). One unit of ACC deaminase activity was expressed as the amount of α-ketobutyrate liberated in ηmol per milligram of cellular protein per hour.

### Identification of Selected Endophytes Based on 16S rRNA Cataloging and Phylogenetic Analyses

All the endophytic isolates from peanuts were processed for isolation of genomic DNA. Genomic DNA was isolated from exponentially growing cultures of the endophytes using a genomic DNA isolation kit (Promega, Madison, WI, United States) as per manufacturer protocol. Additional treatment of lysozyme was given for Gram positive isolates at 65°C for 30 min for isolation of genomic DNA. PCR amplification was performed in a thermal cycler (Takara^*TP*^ 600 Gradient; Takara Inc., Japan) in 20-μl reaction mixtures containing 2.5 μl of 10 × buffer, 1.5 μl of 25 mM MgCl_2_, 2.5 μl of 2 mM dNTPs, 8.55 μl PCR water, 1.0 μl of universal forward primer (100 pmol) 8F (5′-AGA GTTTGATCCTGGCTCAG-3′), 1.0 μl of universal reverse primer (100 pmol) 1492R (5′-ACG GCTACCTTGTTACGACTT-3′), 0.1 μl RNAase 5 U/μl, 0.3 μl Taq-DNA polymerase (5 U/μl) and 2.55 μl of bacterial genomic DNA. The PCR conditions used were: one cycle at 95°C for 5 min; 35 cycles at 94°C for 1 min, 58°C for 1 min and 72°C for 2 min; and with a final extension at 72°C for 10 min. The amplified products were column purified using a PCR purification kit (Promega, Madison, WI, United States).

The column purified products of the near full-length sequences of 16S rRNA were gel purified using a gel purification kit of Qiagen (Qiagen Inc.) and custom sequenced on an ABI 3,730 × l Genetic Analyzer (Applied Biosystems, Foster City) at Sequencher Tech. Pvt. Ltd., Ahmedabad, Gujarat, India. The 16S rRNA gene sequences of the peanut endophytic isolates were processed and deposited in the National Center for Biotechnology Information (NCBI) GenBank database and accession numbers were obtained (Table 1).

**TABLE 1:** Identification, characterization, and plant growth promoting attributes of the endophytic isolates obtained from different peanut plant tissues.

Isolate	*Identity*	Tissue	GenBank Accession Number	NaCl (%)	ACC deaminase	IAA
J1N	*Pantoea agglomerans*	Seed	JX490078	10	ND	+
J2N	*Enterobacter ludwigii*	Seed	JX490058	10	+	+
J6N	*Enterobacter cloacae*	Seed	JX490077	10	+	+
J7N	*Acinetobacter junii*	Seed	JX490076	10	+	+
J8N	*Alcaligenes faecalis*	Seed	JX490057	10	+	+
J9N	*Alcaligenes faecalis*	Seed	JX490075	5	+	+
J11N	*Pseudomonas otitidis*	Seed	JX490074	12.5	+	+
J12N	*Pseudomonas otitidis*	Seed	JX490073	5	+	+
J14N	*Acinetobacter junii*	Seed	JX490072	10	+	+
J17N	*Enterobacter hormaechei*	Seed	JX490071	10	ND	+
J18N	*Brevibacillus brevis*	Seed	JX490070	10	ND	ND
J19N	*Bacillus endophyticus*	Seed	JX490069	12.5	+	+
J20N	*Acinetobacter junii*	Seed	JX490068	10	+	+
J21N	*Bacillus subtilis*	Seed	JX490067	7.5	+	ND
J22N	*Bacillus firmus*	Seed	JX490066	12.5	+	+
J25N	*Bacillus subtilis* subsp. *subtilis*	Seed	JX490065	7.5	ND	ND
J26N	*Bacillus firmus*	Seed	JX490064	10	+	ND
J27N	*Pseudomonas otitidis*	Seed	JX490063	10	+	+
J29N	*Pseudomonas otitidis*	Seed	JX490062	7.5	+	+
J32N	*Pseudomonas aeruginosa*	Seed	JX490061	10	+	+
J40N	*Bacillus subtilis* subsp. *subtilis*	Seed	JX490056	7.5	ND	ND
J45N	*Pseudomonas aeruginosa*	Seed	JX490055	5	+	+
J47N	*Brevibacterium iodinum*	Seed	JX490060	5	ND	ND
J51N	*Pseudomonas otitidis*	Seed	JX490059	5	+	+
SEN9N	*Bacillus subtilis* subsp. *subtilis*	Stem	JX490094	10	ND	ND
SEN12N	*Brevibacterium casei*	Stem	JX490093	7.5	ND	ND
SEN13N	*Staphylococcus arlettae*	Stem	JX490092	5	ND	ND
SEN14N	*Kocuria palustris*	Stem	JX490089	5	ND	ND
SEN15N	*Bacillus tequilensis*	Stem	MW362448	10	+	+
SEN16N	*Brevundimonas diminuta*	Stem	JX490088	5	ND	ND
SEN17N	*Staphylococcus caprae*	Stem	JX490087	5	ND	ND
SEN18N	*Bacillus nealsonii*	Stem	JX490086	5	ND	ND
SEN21N	*Staphylococcus haemolyticus*	Stem	JX490085	5	ND	ND
SEN29N	*Pseudomonas pseudoalcaligenes*	Stem	JX490082	10	+	+
REN12N	*Brevibacterium iodinum*	Root	JX490101	10	ND	ND
REN14N	*Agrococcus casei*	Root	JX490100	10	ND	ND
REN15N	*Brevibacterium avium*	Root	JX490125	10	ND	ND
REN21N	*Staphylococcus capitis*	Root	JX490122	5	ND	ND
REN22N	*Kytococcus sedentarius*	Root	JX490098	5	ND	+
REN24N	*Pseudomonas aeruginosa*	Root	JX490121	10	+	+
REN37N	*Bacillus megaterium*	Root	JX490117	7.5	+	+
REN38N	*Dietzia maris*	Root	JX490116	5	ND	+
REN39N	*Bacillus circulans*	Root	JX490115	10	+	ND
REN40N	*Bacillus subtilis* subsp. *subtilis*	Root	JX490113	10	ND	ND
REN42N	*Bacillus megaterium*	Root	JX490112	10	+	+
REN43N	*Bacillus subtilis* subsp. *subtilis*	Root	JX490111	7.5	ND	ND
REN44N	*Brevibacterium album*	Root	JX490110	5	ND	ND
REN47N	*Pseudoxanthomonas mexicana*	Root	JX490109	10	+	+
REN51N	*Bacillus* sp.	Root	JXAB0 1000000	10	+	+
REN52N	*Paenibacillus xylanilyticus*	Root	JX490107	10	ND	+
REN56N	*Bacillus endophyticus*	Root	JX490106	12.5	+	+
REN59N	*Bacillus firmus*	Root	JX490096	7.5	+	+
REN66N	*Bacillus megaterium*	Root	JX490097	10	+	+
REN67N	*Bacillus megaterium*	Root	JX490105	7.5	+	+
REN68N	*Lysinibacillus boronitolerans*	Root	JX490104	7.5	ND	ND
REN69N	*Bacillus megaterium*	Root	JX490103	7.5	+	+

The identities of the endophytes were obtained by comparing sequences of the isolated 16S rRNA gene with available sequences in the GenBank^1^ using the BLASTn program. Phylogenetic analyses of 55 16S rRNA sequences were performed using MEGA (Molecular Evolutionary Genetics Analysis software) version 6.0 (Tamura et al., 2013) after multiple alignments of the data by CLUSTAL W (Thompson et al., 1994) using progressive alignment method in the same software. Phylogenetic trees were constructed by the UPGMA method using bootstrap (Felsenstein, 1985; 1000 replications) and maximum composite likelihood model in MEGA software ver. 6.0 (Tamura et al., 2013). Then organ/tissue-wise (i.e., seed, stem, and root) relationship was assessed separately with the same software and parameters.

### Germinating Seed Bioassay With Selected Endophytes Having Tolerance to Salinity and ACC Deaminase and IAA Producing Traits

For germinating seed bioassay, 31 endophytic bacterial isolates, having ACC deaminase activity in addition to salinity tolerance, were grown in 25 ml of Luria broth (M575, Himedia) for 48 h in a shaker at 240 rpm at 28°C. The cultures were centrifuged at 10,000 rpm in a refrigerated centrifuge at 4°C. The pellets were suspended in standard nutrient broth (SNB) as described earlier (Gerhardson et al., 1985; Pal et al., 1999) adjusting the OD to 0.2 at 600 ηm. The seeds of peanut (cultivar GG2: moderately resistant to salinity and TG37A: susceptible to salinity), uniform in size and shape, were selected and surface sterilized with 0.1% HgCl_2_ for 3 min, rinsed with sterile water, and then surface sterilized again in 90% alcohol for 3 min and rinsed five times with sterile water to remove traces of alcohol.

The surface sterilized seeds, thus obtained, were placed onto 0.8% sterile water agar (10 seeds in each plate) and incubated at 28°C for 48 h. Three pre-germinated seeds of uniform radicle size were placed onto sterile 0.8% water agar (three replicates for each treatment; three pre-germinated seeds in each plate) amended with 0, 25, 50, and 75 mM NaCl (limit of salinity stress at which peanut seed can germinate but with reduced vigor) and 10 μl of each culture suspended in SNB was placed on each pre-germinated seed and incubated at 28°C for 7 days. The uninoculated respective control received 10 μl of SNB only. The length of the root of each seedling was measured after 7 days and expressed in cm to evaluate the ability of the endophytes in elongation of roots of peanut and compared with roots of respective uninoculated treatments.

### Intrinsic Antibiotic Resistance (IAR) Patterns of Endophytes

The intrinsic antibiotic resistance patterns (IAR) of the endophytes were determined using antibiotics for different groups following Dey et al. (2004), up to 150 μg/ml [0, 25, 50, 100, and 150 μg/ml concentration: β-lactams (Ampicillin-Ap); aminoglycosides (Streptomycin-Sm and Kanamycin-Km); tetracyclines (Tetracycline-Tc); aminocyclitol (Spectinomycin-Spc); others (Chloramphenicol-Cm and Nalidixic acid-Nal)]. Twenty-four-hour-old cultures of endophytes grown in Luria broth at 28°C in a shaker, at 240 rpm, were used to make bacterial spots (5 μl) in triplicates, onto Luria agar (LA) medium plate amended with the antibiotics, as mentioned earlier at different concentrations. The petri plates were incubated at 28°C for 5 days, and the growth on the spots was recorded at 24 h intervals. Finally, the patterns of antibiotic resistance for each endophytic isolate were determined, which was used for tracking the endophytes inside different tissues (root, stem, and seed) of peanut during experiments, to ascertain the nature of colonization.

### Screening of Selected Endophytes for Alleviation of Salinity Stress in Potted Condition

Based on the results obtained in germinating the seed bioassays, a total of 31 isolates of endophytes were evaluated for their capacity to alleviate salinity stress in potted conditions. The experiment was laid in pots, following Dey et al. (2004), having 18′′ diameter and capacity to hold 20 kg of soil (medium black and calcareous, pH 7.9, organic carbon 0.52%, total nitrogen content 0.2125 g/kg, available phosphorus 0.0075 g/kg, and K_2_O 0.120 g/kg). Unsterile soil was used for the experiment. There were a total of 32 treatments, each having six replications. Three replications were used for measuring root and shoot length. The remaining three replications were used for observation after harvest.

Peanut cultivar TG37A (susceptible to salinity), a Spanish Bunch variety, was used in the experiment during the post-rainy season. Pots were kept in the open and watered at alternate days with 2 EC (2.0 dS/m) saline water made by adding the required quantity of NaCl, beginning 15 days after emergence @ 1000 ml of water of 2.0 dS/m salinity. The water with 2.0 dS/m salinity was made by adding NaCl @1.17 g/l. Initial soil salinity was 0.2 dS/m at the time of sowing. Until 15 days of germination, watering was done with normal water only to facilitate uniform germination in all the treatments. Nitrogen at 0.01 g/kg soil as ammonium sulfate and P_2_O_5_ at 0.02 g/kg soil as single super phosphate were applied just before sowing. Each isolate of endophyte was grown overnight in Luria broth. Each broth was centrifuged at 12,000 rpm, washed with phosphate buffer three times, and then pellets were dissolved in 0.1 M phosphate buffer (pH 7.0) and OD was adjusted to 1.2 before being used for pot experiments. An OD of 1.2 was equivalent to 2.0 × 10^8^ CFU/ml.

The seeds for each treatment were soaked for an hour in phosphate saline buffer (PSB) containing the suspension of the endophyte isolates to maintain a population of approximately 10^8^ CFU/seed. Seeds of the control treatment received only phosphate buffer. In each pot, eight seeds (95% germination) were sown at a depth of 5 cm. After germination, five seedlings were maintained in each pot. Root and shoot length was measured at 60 days after emergence, along with proline and total phenol content, in leaf. Plant biomass and pod yield were recorded at harvest.

### Field Trials for Assessing the Role of Endophytes in the Alleviation of Salinity Stress (Natural Saline Soil and Irrigation Water)

A field trial was conducted during the rainy- and post-rainy seasons of 2013 at the Regional Research Station (RRS) of Central Arid Zone Research Institute (CAZRI), Kukma, Bhuj, Gujarat, India. The soil parameters of the site of experiment were: sandy loam to loamy sand; available P as P_2_O_5_: 11–14 kg/ha; available N: 300 kg/ha; K as K_2_O = 360 kg/ha; electrical conductivity (EC): 2.3–5.3 dS/m; organic carbon (OC) = 0.02–0.03; pH = 8.0–8.2; rainfall: 50–250 mm (rainy season); summer temperature ∼48°C; bulk density: 1.2–1.3 g/cc; soil depth: 30–60 cm; water: saline (electrical conductivity of 1.5–2 dS/m).

The physico-chemical properties of the soil were determined by standard procedures. This includes available P as P_2_O_5_ (Olsen, 1954); available N by alkaline permanganate (KMnO_4_) method (Subbiah and Asija, 1956); available K (Jackson, 1974); and organic carbon content by wet digestion method (Walkley and Black, 1934). The electrical conductivity of soil was measured with the help of an EC meter (Model CM-180; Systronics) and expressed as dS/m. Soil pH was measured with the help of a pH meter (Model LI-610) as described by Chopra and Kanwar (1982). The bulk density of the soil was determined using the standard Core Sampler method (Singh, 1980).

In the field, recommended doses of fertilizers (20 kg N/ha as ammonium sulfate; 40 kg P_2_O_5_/ha as single super phosphate, and 60 kg K_2_O/ha as muriate of potash) were used unless mentioned otherwise. Nine endophytes (*Alcaligenes faecalis* J8N, *Pseudomonas otitidis* J11N, *Acinetobacter junii* J20N, *Bacillus firmus* J22N, *Bacillus tequilensis* SEN15N, *Pseudomonas pseudoalcaligenes* SEN29N, *Pseudomonas aeruginosa* REN24N, *Pseudoxanthomonas mexicana* REN47N, and *Bacillus* sp. REN51N) were evaluated with susceptible cultivar TG37A. There were three replicates (design RBD) with 30 cm row-to-row and 10 cm plant-to-plant spacing in 10 rows of 5 m length. The sowing was done during the rainy (first fortnight of July) and post-rainy (first fortnight of February) seasons of 2013. The seeds were coated with charcoal-based (charcoal as a carrier) endophytes cultures (10^9^ –10^10^ CFU/g carrier). Seeds (120 kg/ha) were sown at a depth of 5 cm in rows. A plant population of 480–490 plants/plot was maintained by thinning if required. As rainfall was scanty during the time of post-rainy (no rainfall) and rainy (rainfall 75 mm at the site of the experiment) season, 12 irrigations were applied at weekly intervals as flood irrigation. Out of the 12 irrigations, the first four irrigations used normal water (with no salinity) through tankers to facilitate germination, as the soil was saline. The remaining irrigations were provided with saline water (electrical conductivity of 1.5–2.0 dS/m). At the beginning of the experiment during the post-rainy season, soil EC was 2.3 dS/m. Both pH and EC were monitored at 30 days intervals, beginning at the time of sowing. At 60 days after emergence (DAE; pod filling stage), leaf samples were collected for both physiological (relative water content) and biochemical traits [superoxide dismutase (SOD), ascorbate peroxidase (APX), glutathione reductase (GR), peroxidases, catalases, and contents of H_2_O_2_, total phenol, proline, etc.]. The population densities of the endophytes were measured in different tissues at 45 and 90 days after emergence. The crop was harvested after maturity at 110 days after sowing (DAS) and pod and haulm yield and uptake were recorded. The crop was raised in the same piece of land during the rainy and post-rainy seasons of 2013.

### Field Trials for Assessing the Role of Endophytes in the Alleviation of Salinity Stress at Elevated Salinity

The experiment was conducted with three levels of saline water (normal: 1.5–2 dS/m of salinity; 3 dS/m of salinity and 6 dS/m of salinity) with two cultivars (GG2: moderately resistant and TG37A: susceptible to salinity) in a split-plot design, with three rows of each of the two varieties in 5 m row length in the same plot, with row-to-row and plant-to-plant spacing of 30 and 10 cm, respectively.

There were three replications for each treatment. At the beginning of the experiment, the EC of soil was 2.46 dS/m. The crop was raised with three endophytes (*Bacillus firmus* J22N: seed endophyte; *Bacillus tequilensis* SEN15N: stem endophyte; and *Bacillus* sp. REN51N: root endophyte) keeping an uninoculated treatment during the post-rainy season of 2013, and rainy season of 2013 and 2014 in the same plots in successive seasons. As rainfall was scanty during the time of the post-rainy (no rainfall) and the rainy season (rainfall 75 mm at the site of the experiment), 12 irrigations were applied at a weekly interval as flood irrigation. Out of the 12 irrigations, the first two irrigations were provided with normal water (no salinity) through tankers to facilitate germination, as the soil was saline. All other parameters were as mentioned in the previous experiment. All observations like biochemical traits, population dynamics, pH, and the EC of soil, yield, and biomass were recorded as described earlier.

### Population Densities of Endophytes Inside Plant Tissues

The population densities of inoculated endophytes were determined in root and shoot samples at 45 and 90 days after emergence (DAE), and inside the seed at 90 DAE, in both of the above field experiments based on IARs onto Kings’s B medium amended with appropriate antibiotics. The population was expressed as a log number of cells/g. Five grams each of the root, stem, and seed (developing) were taken for determining the population of the inoculant strains inside the tissues. Roots were gently dipped in sterile tap water to remove the still adhering soil particles. Samples were processed as described earlier for isolation of endophytes. Cycloheximide (100 μg/ml) was also amended in medium to inhibit the fungal growth. The colonies were counted and expressed as a log number of cells/g. Colony morphology and characteristics were also taken into consideration while counting to avoid the counting of the spontaneously growing population.

### Monitoring the Changes in pH and EC of Soil

The changes in pH and EC of soil were monitored at 30 days interval in all the experimental sites by taking soil samples at 0–10 cm in the top layer of soil (three samples each from all three replications of the experimental plots). For determining soil pH and EC, the soil was diluted with distilled water @1:2.5 dilution and pH and EC were determined in a pH (Model: μ pH system; Systronics)/TDS meter (Model CM-180; Systronics).

### Quantification of Plant Physiological and Biochemical Traits

The relative water content (RWC) of a leaf was estimated by recording the fresh, turgid, and dry weight of leaf samples collected from the field following the formula: RWC = [(Fresh wt.-Dry wt.)/(Turgid wt.-Dry wt.)] × 100 (Weatherley, 1950). The content of hydrogen peroxide (H_2_O_2_) was measured through the formation of the titanium-hydro peroxide complex (Rao et al., 1997). One gram leaf material was ground with liquid nitrogen and the finely powdered material was mixed with 10 ml of ice cooled acetone in a cold room. The mixture was filtered through Whatman No. 1 filter paper followed by the addition of 4 ml of titanium reagent and 5 ml of ammonium solution to precipitate the titanium-hydro peroxide complex. The reaction mixture was centrifuged at 12,000 rpm for 15 min at 4°C. The precipitate was dissolved in 10 ml of 2 M H_2_SO_4_ and then re-centrifuged. The absorbance of the supernatant was taken at 415 ηm against blank. Hydrogen peroxide contents were calculated by comparison with a standard curve drawn with a known quantity of hydrogen peroxide. The extract for SOD, APX, GR, POD, and CAT was prepared by first freezing one gram of leaf samples in liquid nitrogen to prevent proteolytic activity followed by grinding with 10 ml extraction buffer (0.1 M phosphate buffer, pH 7.5, containing 0.5 mM EDTA in case of SOD, GR, POD, CAT, and 1 mM ascorbic acid in the case of APX). The extract was passed through four layers of cheesecloth and filtrate was centrifuged for 20 min at 15,000 × *g* and the supernatant was used as an enzyme.

The total SOD (EC 1.15.1.1) activity was measured by the inhibition of the photochemical reduction of NBT by the enzyme (Dhindsa et al., 1981). The 3 ml reaction mixture contained 13.33 mM methionine, 75 μM NBT, 0.1 mM EDTA, 50 mM phosphate buffer (pH 7.8), 50 mM sodium carbonate, 0.1 ml enzyme extract and water to make a final volume of 3.0 ml. The reaction was initiated by adding 2 mM riboflavin (0.1 ml) keeping the tubes under two 15 W fluorescent lamps for 15 min in dark. Illuminated and non-illuminated reaction mixtures without enzyme were used for calibration. The absorbance was recorded at 560 ηm, and one unit of enzyme activity was taken as that amount of enzyme that reduced the absorbance reading to 50% in comparison with tubes lacking enzyme per unit time. The catalase (CAT) (EC 1.11.1.6) activity was assayed by measuring the disappearance of H_2_O_2_ (Aebi, 1984) in a reaction mixture (3 ml) consisting of 0.5 ml of 75 mM H_2_O_2_ and 1.5 ml of 0.1 M phosphate buffer (pH 7) on the addition of 50 μl of diluted enzyme extract. The decrease in absorbance at 240 ηm was observed for 1 min in a UV-visible double beam spectrophotometer.

Enzyme activity was estimated by calculating the amount of H_2_O_2_ that decomposed. The initial and final contents of H_2_O_2_ were calculated by comparison with a standard curve drawn with known concentrations of H_2_O_2_. The peroxidase (POD) (EC 1.11.1.7) activity was measured in terms of increase in absorbance due to the formation of tetra-guaiacol at 470 ηm, and the enzyme activity was calculated as per extinction coefficient of its oxidation product, tetra-guaiacol ε = 26.6/mM/cm (Castillo et al., 1984). The reaction mixture contained 50 mM phosphate buffer (pH 6.1), 16 mM guaiacol, 2 mM H_2_O_2_, and 0.1 ml enzyme extract. The mixture was diluted with distilled water to make up a final volume of 3.0 ml. Enzyme activity was expressed as μmol tetra-guaiacol formed/min/mg protein. The APX (EC 1.11.1.11) activity was assayed by recording the decrease in optical density due to ascorbic acid at 290 ηm (Nakano and Asada, 1981). The reaction was set up in a 3 ml reaction volume containing 50 mM potassium phosphate buffer (pH 7.0), 0.5 mM ascorbic acid, 0.1 mM EDTA, 0.1 mM H_2_O_2_, 0.1 ml enzyme, and water to make up a final volume of 3.0 ml. To this, 0.1 ml of H_2_O_2_ was added to initiate the reaction. The decrease in absorbance was measured spectro-photometrically and the activity was expressed by calculating the decrease in ascorbic acid content using a standard curve drawn with known concentrations of ascorbic acid and expressed as μmol oxidized ascorbate/mg protein/min.

The activity of GR (EC 1.8.1.7) was assayed using the method of Smith et al. (1988). The reaction mixture contained 66.67 mM potassium phosphate buffer (pH 7.5) and 0.33 mM EDTA, 0.5 mM 5,5-dithiobis-(2-nitro) benzoic acid in 0.01 M potassium phosphate buffer (pH 7.5), 66.67 μM NADPH, 666.67 μM oxidized glutathione (GSSG) and 0.1 ml enzyme extract. The mixture was diluted with distilled water to make up a final volume of 3.0 ml. The reaction was initiated by adding 0.1 ml of 20.0 mM GSSG. The increase in absorbance at 412 ηm was recorded spectro-photometrically and the activity was expressed as μmol of GSSG reduced per mg protein per min. Total soluble protein was determined according to the method of Bradford (1976), with bovine serum albumin as standard. The content of proline was determined according to the method by Bates et al. (1973). Leaf tissue (0.2 g) was homogenized with 10 ml of 3% aqueous sulfosalicylic acid and the homogenate was centrifuged at 10,000 rpm for 10 min. Then, 2 ml of supernatant were mixed with 2 ml of glacial acetic acid and 2 ml of acid ninhydrin for 1 h at 100°C. The reaction was terminated by putting the tubes in an ice bath after adding 5 ml toluene into them. The upper phase of the developed color was measured spectrophotometrically at 520 ηm against toluene using UV-Vis double beam spectrophotometer (Specord Bio 200, Analytik Jena, Germany). A standard curve with L-proline was used for the final calculations. The proline content was expressed in mg/g FW (Fresh weight). Total phenol content (TPC) was determined using the method of Zheng and Shetty (2000). A leaf sample (0.1 g) was kept in ethanol (95%, 5 ml) for 48 h for extraction, homogenized, and centrifuged at 13,000 *g* for 10 min. The supernatant (1 ml) was mixed with 95% ethanol (1 ml) and SDW (5 ml). To this, 0.5 ml of 1 N Folin–Ciocalteu reagent was added. After 5 min, 1 ml of 5% Na_2_CO_3_ was added, and the reaction mixture was allowed to stand for 60 min after which the absorbance at 725 ηm was recorded. The calibration curve was prepared with different concentrations of gallic acid (GA) in 95% ethanol. Absorbance values were converted to mM gallic acid equivalent (GAE) /g FW and then expressed as mg/g FW.

### Estimation of K Content in Plant

The oven-dried plant samples collected at harvest were finely ground and dry materials were subjected to tri-acid digestion. The K content in homogenized plant sample was determined using a flame photometer (ELICO; CL378).

### Statistical Analysis

All statistical analyses were performed following Gomez and Gomez (1984) and SPSS package. The data were analyzed using the variance (ANOVA) applicable to the design of the experiment. Mean separations were performed by Tukey’s multiple range test as per experimental need. Differences at *P* ≤ 0.05 were considered significant. The population of bacteria was estimated after the log transformation of raw data and expressed as log CFU/g tissue.

## Results

### Isolation, Characterization, Identification, and Diversity of Endophytes of Peanut

**FIGURE 1:**
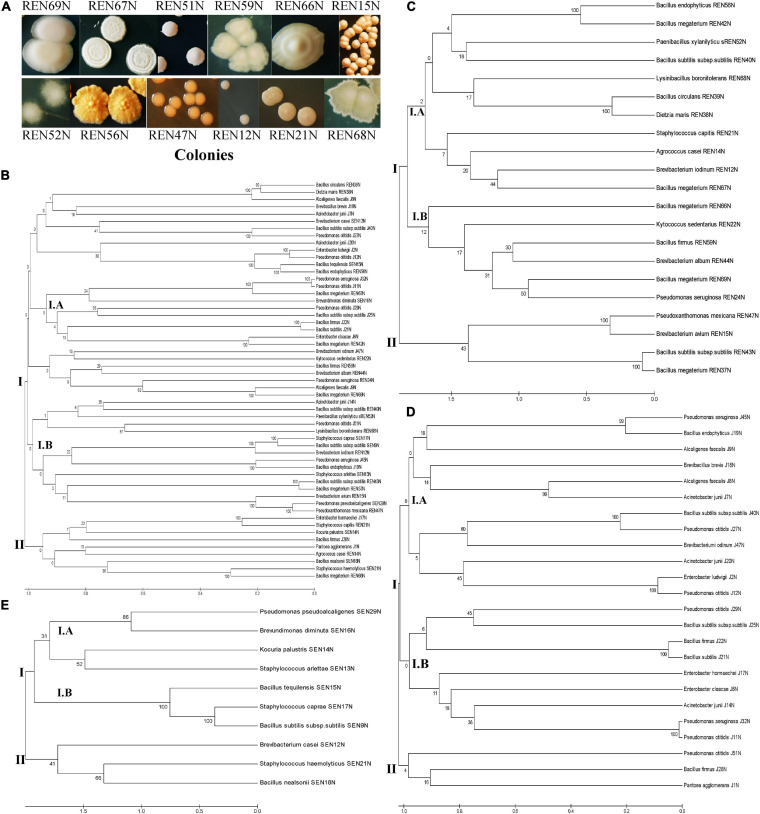
**(A)** Colonies of different root endophytes as appeared on King’s B medium. **(B–E)** Diversity of endophytes of peanut, based on 16S rRNA sequences. Phylogenetic relationships of the endophytes based on 16S rRNA gene sequences. The evolutionary history of 55 endophytic isolates was inferred using the UPGMA method (Tamura et al., 2013). The percentage of replicate trees in which the associated taxa clustered together in the bootstrap test (1000 replicates) is shown under/above the branches (Felsenstein, 1985). The phylogenetic tree was constructed using the UPGMA method with bootstrap test and maximum composite likelihood model in MEGA software ver. 6.0 (Tamura et al., 2013). The tree is drawn to scale in order to infer the phylogenetic relationship. The evolutionary distances were computed using the p-distance method (Nei and Kumar, 2000) and are in the units of the number of base differences per site. The analysis involved 55 nucleotide sequences of bacterial endophytes. Evolutionary analyses were conducted in MEGA software ver. 6.0 (Tamura et al., 2013) using nucleotide substitution including transition and transversion. The evolutionary relationship/diversity among tissues specific (stem, root, and seed; 10, 21, and 24 nucleotide sequences, respectively) endophytes are drawn separately. **(B)** Overall diversity; **(C)** diversity among root endophytes; **(D)** diversity among seed endophytes; **(E)** diversity among stem endophytes.

From different tissues (including the root, stem, and seeds) of peanut, 56 different isolates of endophytes distinct in colony morphology and pigmentation were obtained (Figure 1A) onto King’s B medium. Out of the isolates 22, 10, and 24 were obtained from the root, stem, and seed of peanut (cultivar GG2 and TG3A combined), respectively. The 16S rRNA sequences of all the 56 endophyte isolates were submitted to NCBI GenBank and accession numbers were obtained (Table 1).

The endophytic bacteria were named according to a blast search of the 16S rRNA sequences in the NCBI database. They shared 98.5–100% similarity with the existing 16S rRNA database at NCBI. The endophytes identified so far were represented by 14 different genera (*Bacillus* and *Bacillus* derived, *Pantoea*, *Alcaligenes*, *Enterobacter*, *Acinetobacter*, *Pseudomonas*, *Pseudoxanthomonas*, *Brevibacterium*, *Staphylococcus*, *Kocuria*, *Brevundimonas*, *Agrococcus*, *Dietzia*, and *Kytococcus*, Table 1 and Figure 1B).

Among the total endophytes, *Bacillus* and its derived genera were the most predominant (23 out of 56) followed by *Pseudomonas* (09 out of 56), four each of *Acinetobacter* and *Staphylococcus*, three of *Alcaligenes*, two of *Enterobacter*, and the rest of the genera represented by single isolates. The endophytes capable of colonizing the peanut seed belonged to the genera *Pseudomonas* (07), *Bacillus* and its derived genera (07), *Enterobacter* (03), *Acinetobacter* (04), *Alcaligenes* (02), and *Brevibacterium* (01) (Table 1 and Figure 1C). The seed endophytes of the genera *Enterobacter*, *Acinetobacter*, and *Alcaligenes* could not be detected among the root and stem endophytes of the present pool (Table 1).

Similarly, stem endophytes were represented by six different genera belonging to *Bacillus* and its derived genera (03), *Staphylococcus* (03), and one each from the genus *Pseudomonas*, *Brevibacter*, *Kocuria*, and *Brevundimonas* (Table 1 and Figure 1E). The rest eight genera, among the total 14 genera representing endophytes in peanut in the present pool of organism, could not be obtained. Root endophytes were the most diverse represented by nine different genera (*Bacillus* and its derived-13, *Brevibacterium*-03, and one each of the genus *Agrococcus*, *Staphylococcus*, *Kytococcus*, *Pseudomonas*, *Pseudoxanthomonas*, and *Dietzia*) (Table 1 and Figures 1A–E). *Agrococcus*, *Kytococcus*, *Pseudoxanthoma*, and *Dietzia* could not be found either in the stem or in the seed. Similarly, *Brevundimonas* was found only in the stem. Apart from *Bacillus* and its derived genera, both *Pseudomonas* and *Brevibacterium* were found as common endophytes in all three niches (Table 1 and Figures 1B–E). The pigmentation among the root endophytes was the most diverse, ranging from gummy creamy-white to semi-dry yellowish-orange colonies (Figure 1A). All the endophytes were evaluated for tolerance to salinity *in vitro*. All the 56 endophytic bacteria showed tolerance to NaCl, ranging from 5 to 12.5% (Table 1), a maximum of 12.5% was exhibited by *Pseudomonas otitidis* J11N, *Bacillus endophyticus* J19N, *Bacillus firmus* J22N, and *Bacillus endophyticus* REN56N (Table 1).

The diversity of 55 endophytes was studied by drawing a phylogenetic tree, tissue-wise as well as in totality (Figures 1B–E). The phylogenic tree obtained based on 16S rRNA sequences of all the endophytes was bifurcated into two lineages *viz*., lineage I and Lineage II (Figure IB). The lineage I covered most of the endophyte species, which were clustered into two sub-clusters (Cluster I.A and Cluster I.B). Similarly, the phylogenetic trees of root colonizing (Figure 1C), seed colonizing (Figure 1D), and stem colonizing (Figure 1E) endophytes were differentiated into two further sub-clusters.

All the isolates were evaluated for the presence of ACC deaminase and IAA production capacities. While 31 endophytes exhibited ACC deaminase activity (18 seed-, two stem- and 11 root- endophytes), 33 produced IAA (18 seed-, 2 stem-, and 13 root- endophytes) (Table 1), 28 endophytes were common in both the groups.

**FIGURE 2:**
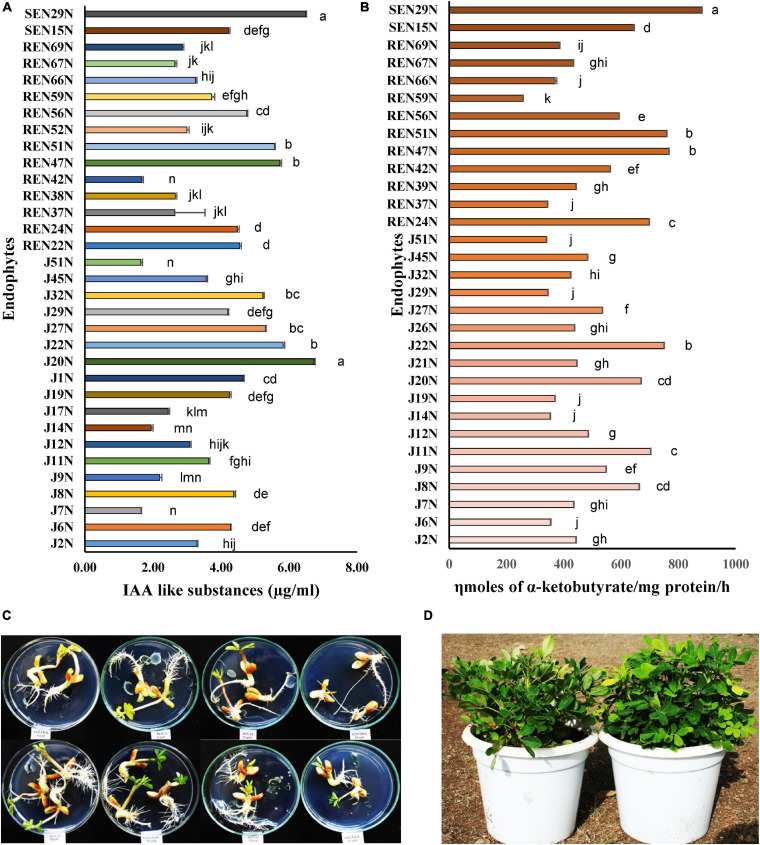
Quantification of plant growth promoting substances by selected endophytes of peanut. **(A)** Indole acetic acid like substances (μg/ml); **(B)** ACC deaminase activity (ηmoles α-ketoglutarate/mg protein/h). Values are mean of three replications ± SE. Data followed by the same letter (s) do not differ significantly at *P* = 0.05 according to Tukey’s multiple range test. LSD_0.05_ for IAA and ACC deaminase among the treatments are 0.494 and 38.12, respectively. **(C)** *In vitro* germinating seed bioassay: effect of inoculation of *Bacillus* sp. REN 51N on root growth and development of peanut (cv. TG37A) seedlings. Observation after 72 h of incubation at 28 ± 2°C; **(D)** impact of inoculation of endophytes on alleviation of salinity stress in peanut (cv. TG37A) in pots; left: uninoculated control; right: inoculated with *Bacillus firmus* J22N.

The ACC deaminase and IAA producing capacities of the endophytes were also quantified. The ACC deaminase activity ranged from 259 to 883 ηmol α-ketobutyrate/mg protein/h (Figure 2B). Endophytes with high ACC deaminase activity were *Pseudomonas pseudoalcaligenes* SEN29N (stem endophyte), *Bacillus* sp. REN51N (root endophyte) and *Bacillus firmus* J22N (seed endophyte) with 883, 760, and 751 ηmol α-keto-butyrate/mg protein/h, respectively (Figure 2B). Similarly, the production of IAA ranged from 1.65 μg/ml to 6.74 μg/ml (Figure 2A), with high production capacity in *Acinetobacter junii* J20N (6.74 μg/ml), *Pseudomonas pseudoalcaligenes* SEN29N (6.49 μg/ml), and *Bacillus firmus* J22N (5.82 μg/ml).

### Germinating Seed Bioassay

**FIGURE 3:**
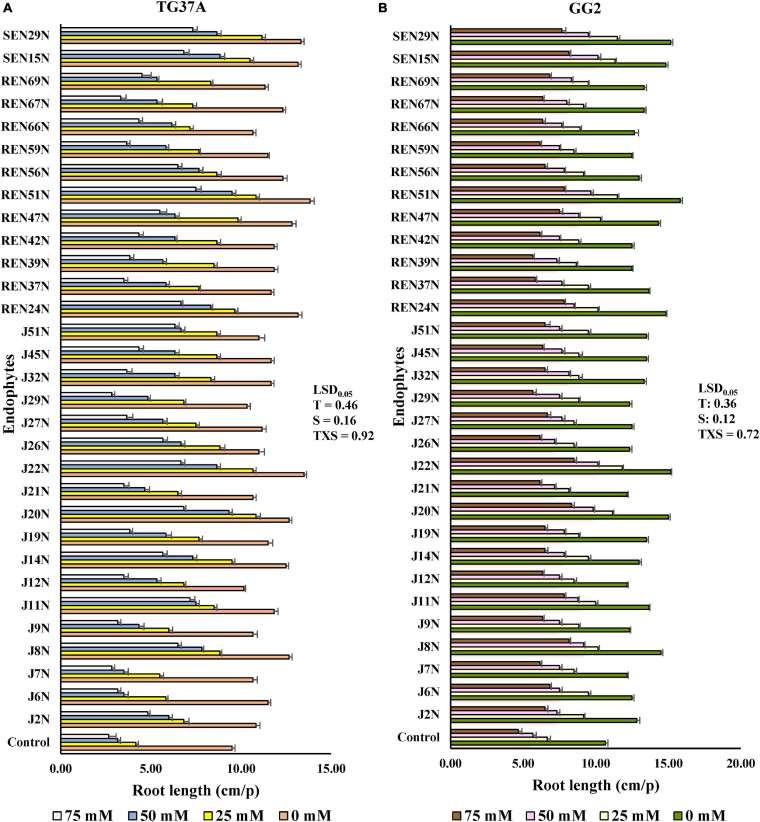
*In vitro* germinating seed bioassay: effect of endophytes and different levels of NaCl on the root growth of peanut seedlings. **(A)** Cultivar TG37A (susceptible) and **(B)** GG2 (moderately resistant) at 28 ± 2°C (data mean of three replications ± SE, three germinating seeds in each replication). LSD_0_._05_ for treatment (T), salinity (S), and TXS are 0.46, 0.16, and 0.92, respectively for cultivar TG37A. Similarly, LSD_0_._05_ for treatment (T), salinity (S), and TXS are 0.36, 0.12, and 0.72, respectively for cultivar GG2.

The germinating seed bioassay was performed with two genotypes of peanut viz. GG2 (moderately resistant to salinity) and TG37A (sensitive to salinity) *in vitro* with the inoculation of 31 endophytes at three levels of salinity (25, 50, and 75 mM of NaCl), keeping a control without NaCl. Inoculation of most of the endophytes significantly improved root growth of TG37A across different levels of salinity, except one at 25 mM, three at 50 mM, 12 at 75 mM of salinity, and seven endophytes with no NaCl supplied (Figure 3A).

The maximum improvement in root length was achieved with inoculation of *Bacillus* sp. REN51N, followed by *Bacillus firmus* J22N and *Pseudomonas pseudoalcaligenes* SEN29N, with an improvement of 45.6, 42.1, and 40.3% over uninoculated control when NaCl was not applied (Figures 2C, 3A). With the increase in salinity, the impact of endophytes became more pronounced. At 25 mM of salinity, the root length of TG37A, a salinity sensitive cultivar, was improved by a maximum of 1.7 fold (4.17 cm in control to a maximum of 11.17 cm in *P. pseudoalcaligenes* SEN29N). This was followed by *Bacillus* sp. REN51N and *Acinetobacter junii* J20N (1.6 fold) and *Bacillus firmus* J22N (1.56 fold) (Figure 3A). However, at 50 and 75 mM of NaCl, improvement in root length was 0.1 fold to 2.0 fold and 0.06 fold to 1.81 fold, respectively (Figure 3A) and a number of endophytes failed to improve root length. Consistently across the salinity, four endophytes *B. firmus* J22N, *Bacillus* sp. REN51N, *B. tequilensis* SEN15N, and *P. pseudoalcaligenes* SEN29N significantly enhanced the root length of salinity sensitive TG37A peanut cultivar (Figure 3A).

In contrast to TG3A, in the moderately resistant genotype GG2, the reaction to salinity was different, and the decrease in root length was not as much as that obtained with sensitive genotype TG37A, across salinity. All the endophytes significantly improved the root length of GG2 at 0, 25 and 50 mM NaCl as compared to the uninoculated control (Figure 3B). However, at 75 mM of NaCl concentration, all endophytes except *Pseudomonas otitidis* J29N and *Bacillus circulans* REN39N significantly enhanced root length (Figure 3B). In the case of GG2 cultivar, inoculation of *B. firmus* J22N, *Bacillus* sp. REN51N, and *B. tequilensis* SEN15N, consistently and significantly enhanced root length across all levels of salinity as compared to the uninoculated control (Figure 3B).

### Alleviation of Salinity Stress in Potted Conditions by Endophytic Bacteria

**FIGURE 4:**
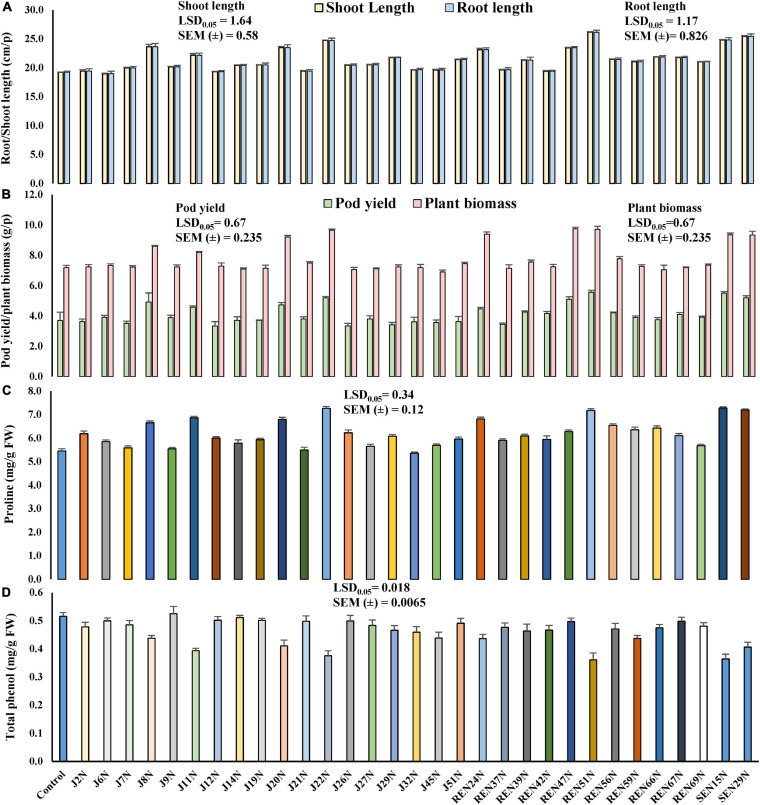
Evaluation of the effect of endophytes in the alleviation of salinity stress, growth, and yield of peanut plants (cv. TG37A) in potted conditions (3.5 dS/m of soil salinity at harvest, irrigated with 2 dS/m saline water made with NaCl) in potted conditions. **(A)** Root and shoot length; **(B)** pod yield and plant biomass; **(C)** proline content; **(D)** phenol content. Data mean of three replications, data with the same letter(s) do not differ significantly at *P* = 0.05 according to Tukey’s multiple range tests.

All the 31 ACC deaminase producing endophytic bacteria were screened further in potted conditions during the post-rainy season of 2012, with an application of a measured quantity of saline irrigation water (electrical conductivity 2.0 dS/m made with NaCl) with the sensitive cultivar of peanut, TG37A. At 60 DAE, the shoot length of TG37A was enhanced significantly with the application of endophyte isolates like J8N, J11N, J20N, J22N (Figure 2D), J29N, J51N, REN24N, REN39N, REN47N, REN51N, REN56N, REN59N, REN66N, REN67N, REN69N, SEN16N, and SEN29N (Figure 4A) as compared to uninoculated control, at the same level of salinity.

Root length also increased significantly due to the inoculation of J8N, J11N, J20N, J22N, J29N, REN24N, REN51N, REN66N, REN67N, REN69N, SEN15N, and SEN29N (Figure 4A) as compared to control. The maximum root and shoot length was obtained with the inoculation of *Bacillus* sp. REN51N. However, at harvest, the inoculation of nine endophytic isolates only viz. J8N, J11N, J20N, J22N, REN24N, REN47N, REN51N, SEN16N, and SEN29N significantly improved plant biomass and pod yield at 3.5 soil EC (3.5 dS/m) (Figure 4B) over control. The maximum pod yield was obtained with the inoculation of root endophyte, *Bacillus* sp. REN51N followed by *B. tequilensis* SEN15N, *P. pseudoalcaligenes* SEN29N and *B. firmus* J22N (Figure 4B). However, maximum enhancement in biomass was noticed with the inoculation of *Pseudoxanthomonas mexicana* REN47N followed by *Bacillus* sp. REN51N and *B. firmus* J22N. The accumulation of phenol and proline in the leaves was also measured at 60 DAE. With the inoculation of endophytes, accumulation of proline as osmoprotectant was improved significantly from 5.45 mg/g FW in control to 7.28, 7.27, 7.20, and 7.17 mg/g FW with *B. tequilensis* SEN15N, *P. pseudoalcaligenes* SEN29N, *B. firmus* J22N, and *Bacillus* sp. REN51N, respectively (Figure 4C), an increase of 33.58, 33.39, 32.11 and 31.56%, respectively over control. Inoculation of all other isolates of endophytes except J14N, J19N, J21N, J45N, J6N, J7N, J9N, REN37N, REN42N, and REN69N significantly improved proline accumulation in leaf in response to salinity (Figure 4C).

The phenol content, an indicator of stress, in the leaves decreased with the inoculation of endophytes, providing succor to the plants in salinity stress. The phenol content varied from 0.516 mg/g FW in control to 0.361 mg/g FW in the case of *Bacillus* sp. REN51N, a decrease of 30.0% (Figure 4D) followed by *B. tequilensis* SEN15N (0.364 mg/g FW) and *B. firmus* J22 (0.376 mg/g FW) (Figure 4D). In general, there was a reduction in the accumulation of phenol with the inoculation of endophytes. Significant reduction in phenol content in the leaf of salinity sensitive peanut cultivar TG3A was achieved with the inoculation of 21 endophytic bacterial isolates (J11N, J20N, J22N, J27N, J29N, J2N, J32N, J45N, J7N, J8N, J9N, REN24N, REN37N, REN39N, REN42N, REN51N, REN56N, REN59N, REN66N, REN69N, SEN15N, and SEN29N), while all other endophytes performed at par that of uninoculated control (Figure 4D).

### Assessing the Role of Endophytes in the Alleviation of Salinity Stress (Natural Saline Soil and Irrigation Water) in Field Conditions

Nine endophytes were selected for further evaluation in fields under natural conditions of water and soil salinity. This was based on the performance of endophytes in *in vitro* germinating seed bioassay, ACC deaminase activity, IAA production capacity, and significant enhancement in the pod and biomass yield of salinity sensitive peanut cultivar TG37A in potted conditions in salinity stress. These were *Alcaligenes faecalis* J8N, *Pseudomonas otitidis* J11N, *Acinetobacter junii* J20N, *Bacillus firmus* J22N, *Bacillus tequilensis* SEN15N, *Pseudomonas pseudoalcaligenes* SEN29N, *Pseudomonas aeruginosa* REN24N, *Pseudoxanthomonas mexicana* REN47N, and *Bacillus* sp. REN51N. The experiment was conducted during the rainy and post-rainy seasons of 2013 with these endophytes. The endophytic bacteria were applied to the field as seed inoculation.

#### Monitoring pH and EC

**FIGURE 5:**
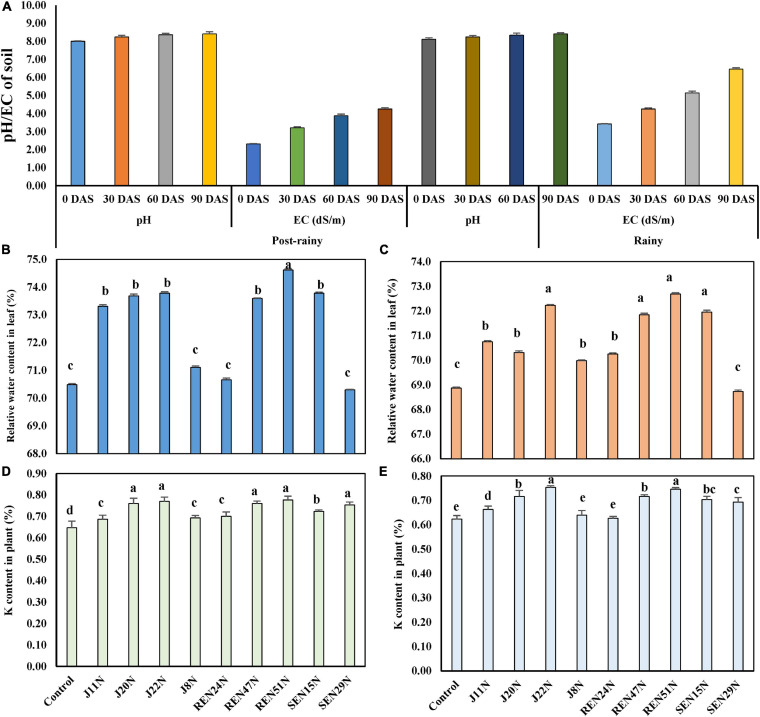
Evaluation of endophytes in field conditions during the post-rainy and rainy season of 2013 with saline irrigation water (1.5–2.0 dS/m salinity) and TG37A. **(A)** development of pH and EC (salinity) in soil; **(B)** influence of endophytes on RWC in peanut leaves during the post-rainy season and **(C)** during rainy season; **(D)** modulation of K uptake by endophytes during rainy season; **(E)** modulation of K uptake by endophytes during the post-rainy season. Data are the mean of three replications, data with the same letter (s) do not differ significantly at *P* = 0.05 according to Tukey’s multiple range test.

The pH and EC of soil at the beginning of the experiment were 8.0 and 2.31 dS/m (Figure 5A) during the post-rainy season. Both the parameters were monitored at 30 days intervals. The soil pH increased with the application of saline irrigation water (1.5–2.0 dS/m of EC), from 8.0 to 8.24, 8.36, and 8.41 at 30, 60, and 90 DAS, respectively. The EC of the soil also increased from 2.31 dS/m to 3.21, 3.87, and 4.25 dS/m at 30, 60, and 90 DAS (Figure 5A), respectively. Similarly, as the same plots were used for the experiment during the rainy season as well, the EC of the soil increased sharply from 3.42 at the beginning to 4.25, 5.14, and 6.46 dS/m at 30, 60, and 90 DAS, respectively (Figure 5A), though there was little effect on the change in pH.

#### Relative Water Content

The relative water content (RWC%) at mid-day was also measured 60 days after the emergence of the crop, in both seasons. Inoculation of endophytes like *B. firmus* J22N (73.69%), *Bacillus* sp. REN51N (74.62%), *B. tequilensis* SEN15N (73.78%), *Acinetobacter junii* J20N (73.69%), *Pseudoxanthomonas mexicana* REN47N (73.59%), and *P. otitidis* J11N (73.31%), significantly improved the relative water content in the leaf of peanut cultivar TG37A over uninoculated control (70.49%) (Figure 5B) during the post-rainy season. All other endophytes performed at par to that of the control during the post-rainy season. During the rainy season, because of the increased salinity due to scanty rainfall, the RWC of control plants was 68.87%. As compared to control, the relative water content improved significantly with the application of all endophytes except *P. pseudoalcaligenes* SEN29N (Figure 5C). While it was maximum in the case of *Bacillus* sp. REN51N (72.69%), the RWC was maintained at 72.23, 71.95, 71.84, 70.74, and 70.31%, respectively by *B. firmus* J22N, *B. tequilensis* SEN15N, *Pseudoxanthomonas mexicana* REN47N, *P. otitidis* J11N, and *A. junii* J20, respectively (Figure 5C).

#### Modulation in Potassium Uptake

There was modulation in the uptake of key nutrients in salinity stress, i.e., potassium. During the rainy season, the uptake of K was improved significantly with the application of all the endophytes over uninoculated control (Figure 5D) in the plants of salinity sensitive peanut (cultivar TG37A), ranging from 6.18 to 20.09% improvement over control. The maximum uptake of K was noticed with the application of *Bacillus* sp. REN51N (0.777%) followed by *B. firmus* J22N (0.770%), *A. junii* J20N (0.760%) and *Pseudoxanthomonas mexicana* REN47N (0.760%) as compared to control (0.647%). With comparatively less salinity during the post-rainy season, the uptake of potassium was also less as compared to the rainy season. There was a significant improvement in the uptake of K with the inoculation of endophytes, except for *A. faecalis* J8N, and *P. aeruginosa* REN24N (Figure 5E). The maximum uptake of K was noticed with the inoculation of *B. firmus* J22N (0.753%), as compared to control (0.623%), with an increase of 20.8%, followed by *Bacillus* sp. REN51N (0.747%), with an increase of 19.9%.

#### Population Densities of Endophytes Inside Different Tissues of Peanut

**FIGURE 6:**
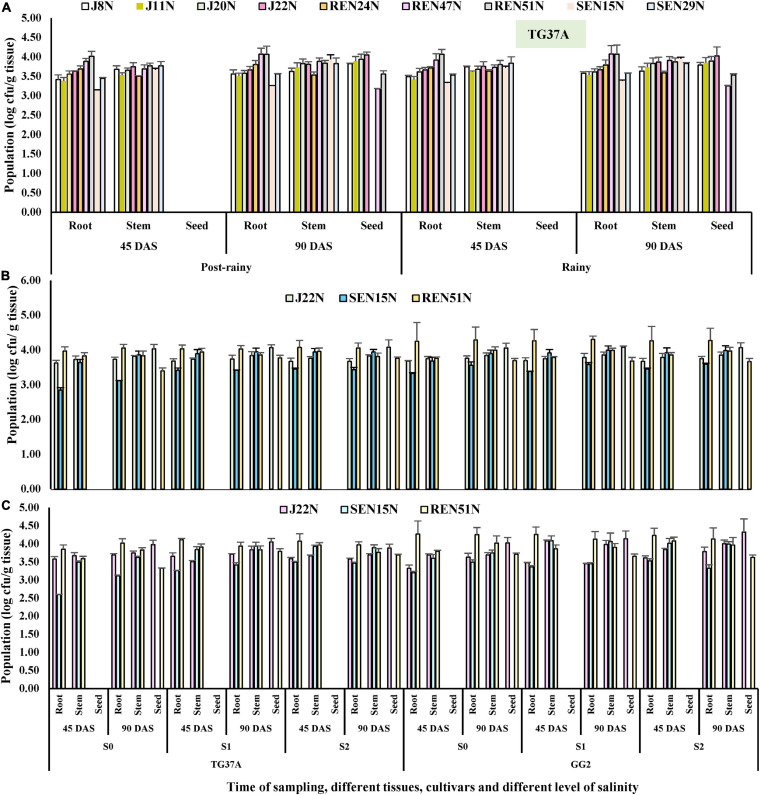
Population densities (after log transformation) of different endophytes inside different tissues of peanut at 45 and 90 days after sowing determined on the basis of intrinsic antibiotic resistance patterns (IAR). **(A)** Population of nine endophytes inside different tissues of TG37A when cultivated at normal saline irrigation water and soil, **(B)** population densities of three endophytes inside peanut during the rainy season. **(C)** Population densities during the post-rainy season.

The population of all the endophytes (root-, stem-, and seed- colonizing) was monitored inside the tissues of the peanut plants at 45 and 90 DAS, using the IARs of each isolate (Table 2) during the post-rainy and rainy seasons. It was observed that seed endophytes viz. J8N, J11N, J20N, and J22N gave population densities of log3.41, log3.38, log3.56, and log3.63 CFU/g root and log3.68, log 3.53, log3.65, and log3.75 CFU/g stem, respectively at 45 DAS (Figure 6A) during the post-rainy season. However, at 90 DAS, the same seed endophytes started proliferating inside the growing seeds as evident from the population of log3.79, log3.85, log3.89, and log4.03 CFU/g seed in the case of J8N, J11N, J20N, and J22N, respectively (Figure 6A). The population of the same endophytes improved marginally in root and stem tissues over their population at 45 DAS. *Bacillus firmus* J22N was the most aggressive colonizer in all three tissues as compared to other seed endophytes. Seed bacterization of peanuts with root endophytes (REN24N, REN4N, and REN51N) resulted in colonization inside the root and stem. Though REN24N failed to colonize seed tissues, both REN47N and REN51N could colonize all three tissues, as evident from their population (Figure 6A). Among the root endophytes, REN51N was the most aggressive colonizer in all three tissues. In the case of stem endophytes viz. SEN15N and SEN29N, both failed to colonize inside the seed tissues (Figure 6A), which gave population densities of log3.15, and log3.45 CFU/g root and log3.66 and log3.77 CFU/g stem at 45 DAS (Figure 6A), respectively. However, at 90 DAS, the population of these endophytes improved further at log3.26 and log3.56 CFU/g in root and log3.93 and log3.83 CFU/g in stem tissues, respectively. With the increase in salinity during the rainy season, the population densities of all the endophytes improved further, with few exceptions (Figure 6A). Results indicated that while the population of all seed endophytes increased gradually from 45 DAS to 90 DAS in all three tissues, stem endophytes viz. SEN15N and SEN29N also failed to colonize seed tissues in this season (Figure 6A). Two of the three root endophytes viz. REN47N and REN51N colonized all three tissues during the rainy season. Among the root endophytes, *Bacillus* sp. REN51N was the most aggressive colonizer, as evident from its population of log4.06 and log4.07 CFU/g root at 45 and 90 DAS as compared to the other two root colonizers (Figure 6A).

**TABLE 2:** Effect of endophytes on growth, yield, and modulation in biochemical parameters of peanut plants (cv. TG37A) in field grown with saline water (electrical conductivity: 1.5–2.0 dS/m; total 12 irrigations including eight saline irrigations) at Bhuj during the rainy and post-rainy seasons of 2013.

Treatments	IAR patterns	PY (kg/ha)		HY(kg/ha)		Total phenol (mg/g FW)*		Proline (mg/g FW)*	
		Post-rainy	Rainy	Post-rainy	Rainy	Post-rainy	Rainy	Rainy	Post-rainy
Control	-	2815	1733	4296	2672	0.546^a^	0.662^a^	6.12^d^	5.54^f^
J8N	Ap^150^Km^25^Sm^100^Spc^150^Cm^150^Tc^150^	2683	1819	4247	2516	0.398^b^	0.631^b^	6.21^d^	5.13^g^
J11N	Ap^150^Nal^50^Sm^25^Spc^50^Cm^150^Tc^150^	3095	1881	4642	3190	0.356^bc^	0.588^c^	7.07^bc^	5.99^e^
J20N	Km^150^Spc^100^Tc^25^	2733	1960	4214	2854	0.339^bc^	0.630^b^	7.43^ab^	6.12^de^
J22N	Ap^25^Spc^25^Cm^50^	2848	1692	4214	2479	0.342^bc^	0.536^d^	7.92^ab^	6.62^ab^
REN24N	Ap^150^Nal^50^Sm^25^Spc^150^Cm^150^Tc^150^	2733	1677	4148	2792	0.511^a^	0.667^a^	7.47^ab^	5.31^fg^
REN47N	Sm^150^Spc^150^	3243	2036	4609	3294	0.315^c^	0.585^c^	6.80^c^	6.35^bcd^
REN51N	Ap^150^Spc^25^Cm^100^	2683	1624	4214	2548	0.318^c^	0.590^c^	7.61^*a*^	6.84^ab^
SEN15N	Nal^150^Spc^150^Cm^50^	3473	1987	5531	3417	0.380^bc^	0.567^c^	7.88^*a*^	6.48^bc^
SEN29N	Ap^50^Nal^50^Cm^150^Tc^150^	2733	1605	4148	2575	0.557^a^	0.583^c^	7.92^a^	6.26^cde^
CV (%)		16.3	16.5	16.50	17.5	8.60	2.08	3.40	2.46
CD (0.05)	-	493	200	309	363	–	–	–	–

*Values are the mean of three replications.

*Data in the same column followed by the same letter(s) do not differ significantly at P = 0.05 according to Tukey’s multiple range test.*

#### Modulation in the Production of ROS Scavenging Enzymes

**FIGURE 7:**
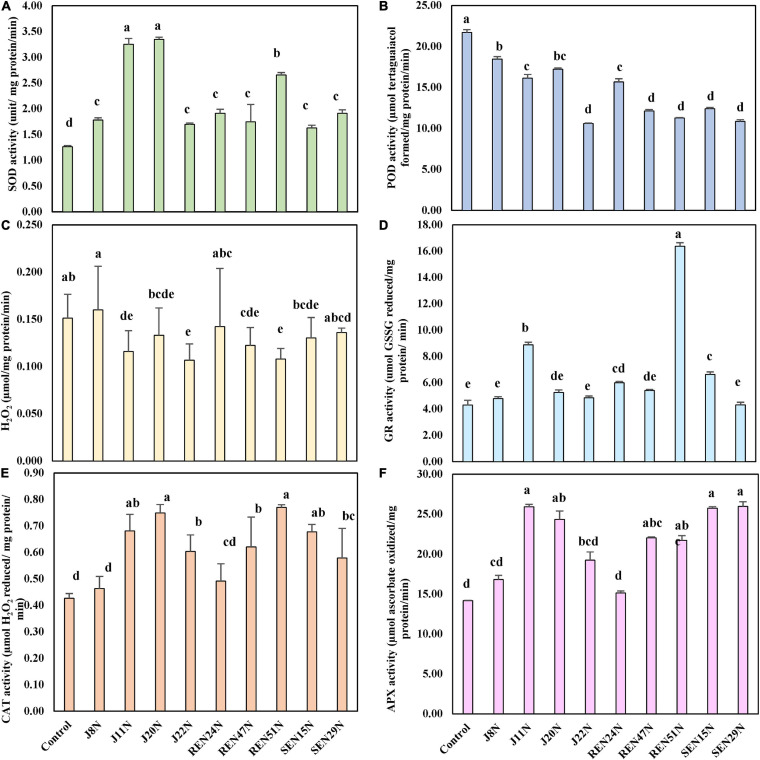
Modulation in the production of ROS scavenging enzymes in peanut (cv. TG37A) in salinity stress by endophytic bacteria in field grown with saline water (electrical conductivity: 1.5–2.0 dS/m; total 12 irrigations including eight saline irrigations) at Bhuj in field condition during post-rainy season of 2013: **(A)** SOD; **(B)** POD; **(C)** H_2_O_2_; **(D)** GR; **(E)** CAT; and **(F)** APX. Data mean of three replications, data with the same letter (s) do not differ significantly at *P* = 0.05 according to Tukey’s multiple range test.

To evaluate the biochemical changes that might take place inside the peanut plant due to the alleviation of salinity stress by the endophytes, different biochemical parameters like the scavenging enzymes involved in degrading ROS generated inside the plant tissues in response to stresses, were studied. The enzymes studied include CAT, SOD, GR, APX, and lipid POD. The content of H_2_O_2_ was also determined. The application of endophytes modulated the level of production of CAT, GR, APX, POD, SOD, and H_2_O_2_ in saline conditions. During the post-rainy season, general scavengers of ROS like CAT, APX, GR, and SOD increased with the imposition of salinity but increased further with the application of endophytes. However, production of POD increased with salinity but decreased with inoculation of endophytes. In the case of SOD activity, there was a significant enhancement in SOD activity when inoculated with endophytes (Figure 7A), with an increase as high as 2.5 fold over the control during the post-rainy season. Maximum SOD activity was obtained with the inoculation of J20N followed by J11N and REN51N (Figure 7A). In the case of CAT, except for those inoculated with J8N and REN24N, there was a significant increase in the production of CAT (range: 34.9–79%) due to the inoculation of the rest of the endophytes with a maximum activity of 0.77 μmol H_2_O_2_ reduced/mg protein/min in case of REN51N as compared to control (0.43 μmol H_2_O_2_ reduced/mg protein/min) (Figure 7E).

The production of APX was also enhanced significantly with the application of endophytes to the control, when inoculated with J11N, J20N, REN47N, REN51N, SEN15N, and SEN29N (Figure 7F). Consequently, the activity of glutathione reductase (GR) was also enhanced significantly, only because of inoculation of REN47N, SEN15N, J11N, and REN51N (Figure 7D). Maximum enhancement of GR activity was noticed when inoculated with REN51N (Figure 7D), with an increase of more than fourfold over the control.

**FIGURE 8:**
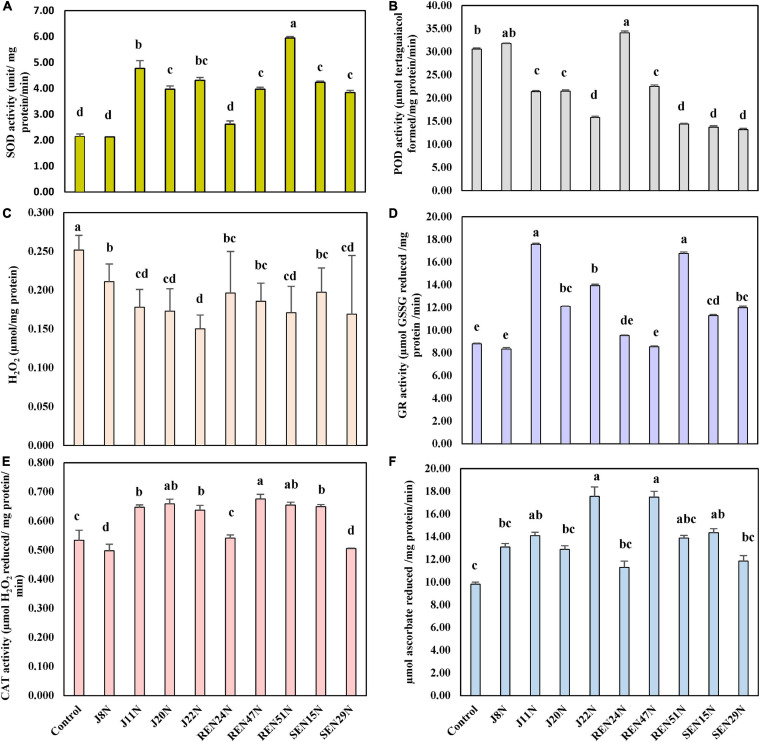
Modulation in the production of ROS scavenging enzymes in peanut (cv. TG37A) in salinity stress by endophytic bacteria in field grown with saline water (electrical conductivity: 1.5–2.0 dS/m; total 12 irrigations including eight saline irrigations) at Bhuj in field condition during rainy season of 2013, **(A)** SOD; **(B)** POD; **(C)** H_2_O_2_; **(D)** GR; **(E)** CAT; and **(F)** APX. Data mean of three replications, data with the same letter (s) do not differ significantly at *P* = 0.05 according to Tukey’s multiple range test.

As a result of the activities of SOD, CAT, APX, and GR, the activities of POD were also reduced significantly when inoculated with all the endophytes, with a maximum reduction in the case of J22N, to almost half (21.7 μmol tetraguaiacol formed/mg protein/min in control, to 10.6 μmol tetraguaiacol formed/mg protein/min in J22N treatment) (Figure 7B). There was less production of stressors because of better scavenging of ROS by enhanced production of CAT, GR, APX, and SOD, there was significantly less accumulation of H_2_O_2_ when inoculated with J20N, J11N, J22N, REN47N, and REN51N (Figure 7C). During the rainy season, with the increase in the salinity in the soil, plants experienced more stress. As a result, the activities of all ROS scavenging enzymes increased during the rainy season in the peanut leaves, except for APX (Figures 8A–F), as there was a further increase in the activities of ROS scavenging enzymes with the inoculation of endophytes.

The seed bacterization of peanut with endophytes significantly increased activities of SOD (except J8N and REN24N; Figure 8A), CAT (except J8N, SEN29N, and REN24N; Figure 8E), APX (except J8N, J20N, SEN29N, and REN24N; Figure 8F) and GR (except J8N, REN24N, and REN47N; Figure 8D). These results indicate that there was a significant reduction in the activities of POD with the inoculation of endophytes like J11N, J20N, J22N, SEN15N, SEN29N, REN47N, and REN51N (Figure 8B).

#### Modulation in the Accumulation of Phenol and Proline

With the imposition of salinity stress, there was a significant enhancement in the accumulation of osmoprotectant and proline in the leaf when inoculated with endophytic bacteria. During the post-rainy season, when the experiment was initiated, the build-up of salinity was less. There was comparatively less accumulation of proline as an osmoprotectant, as compared to that accumulated during the rainy season with an increased level of salinity (Table 2). During the post-rainy season, inoculation of endophytes enhanced the accumulation of proline significantly, except for REN24N over the control (Table 2).

The maximum accumulation of proline was noticed with the inoculation of REN51N (6.84 mg/g FW) followed by J22N (6.62 mg/g FW) and SEN15N (6.48 mg/g FW) (Table 2). It is evident that with the increase in salinity during the rainy season, the inoculation of endophytes like J11N, J20N, J22N, REN24N, REN47N, REN51N, SEN15N, and SEN29N significantly enhanced the accumulation of proline as compared to the control and J8N (Table 2) during the rainy season. The maximum accumulation of proline was noticed with the inoculation of SEN29N (7.92 mg/g FW) followed by SEN15N (7.88 mg/g FW) and REN51N (7.61 mg/g FW). With the accumulation of phenol and the increase in salinity from the post-rainy to the rainy season, there was an increase in the accumulation of phenol content in the leaves (Table 2). However, due to the inoculation of endophytic bacteria, the accumulation of phenol reduced significantly. This significant reduction in the accumulation of phenol was noticed with the seed bacterization with J8N, J11N, J20N, J22N, REN47N, REN51N, and SEN15N during the post-rainy season. Maximum reduction was noticed with the inoculation of REN47N (42.30%) followed by RE51N (41.76%) and J20N (37.91%) over uninoculated control (Table 2). During the rainy season with the increase in salinity, the scenario changed drastically and there was 21.24% more accumulation of phenol in the control treatment. However, with the application of endophytes, there was a reduction in phenol accumulation from 4.68% with J8N to 19.03% with the inoculation of J22N (Table 2). REN24N performed at par with the control.

#### Impact on Pod and Haulm Yield of Peanut

The endophytes were evaluated for two successive seasons (post-rainy followed by rainy) with natural soil and irrigation water salinity. The pod and haulm yield of peanut (cultivar TG37A) was recorded after harvest. It was observed that due to less soil salinity at harvest (4.25 dS/m of salinity) during the post-rainy season, the pod and haulm yield of peanut was much higher (Table 2) as compared to that obtained during the rainy season with soil salinity of 6.46 dS/m at 90 DAS. Whereas pod yield ranged from 2683 kg/ha to 3473 kg/ha during the post-rainy season, it ranged from 1605 kg/ha to 2036 kg/ha during the rainy season (Table 2). There was a reduction of 35.36–41.38% of yield during the rainy season, because of the build-up of salinity, with different endophytes. However, due to inoculation of endophytes, there was a significantly higher pod yield of TG37A when inoculated with *B. tequilensis* SEN15N (3473 kg/ha) compared to the control (2815 kg/ha), with an increase of 23.37% (Table 2).

All other treatments performed at par to that of the control. However, there was an increase in yield due to the inoculation of *P. otitidis* J11N by 9.94% and with *Pseudoxanthomonas mexicana* REN47N by 15.20%. In terms of haulm yield, a significant increase was noticed with the inoculation of SEN15N (5531 kg/ha), J11N (4642 kg/ha), and REN47N (4609 kg/ha) over the control (4296 kg/ha), an increase to the tune of 28.75%, 8.05%, and 7.28%, respectively (Table 2). All other endophytes performed at par that of control. During the rainy season, the level of pod and haulm yield was much lower. With the inoculation of REN47N (2036 kg/ha), SEN15N (1987 kg/ha), and J20N (1960 kg/ha) only, significantly higher pod yield was obtained over control (1733 kg/ha). At the same time, haulm yield enhanced significantly with the application of SEN15N, REN47N, and J11N with an increase of 27.88, 23.28, and 19.39%, respectively, over the control (Table 2).

### Assessing the Role of Endophytes in the Alleviation of Salinity Stress at Elevated Levels of Salinity

A separate experiment was conducted with one of each seed- (*Bacillus firmus* J22N), stem- (*Bacillus tequilensis* SEN15N), and root- (*Bacillus* sp. REN51N) endophyte of peanut plants, with one variety each of a sensitive (TG37A) or moderately resistant (GG2) category. Three levels of irrigation water salinity were used (available saline water of 1.5–2.0 dS/m; 3.0 dS/m, and 6 dS/m). The initial average soil EC and pH were 2.44 dS/m and 8.14, respectively. The same plot was used for experimentation during the post-rainy season of 2013 and the rainy season of 2013 and 2014.

#### Monitoring the pH and EC

**FIGURE 9:**
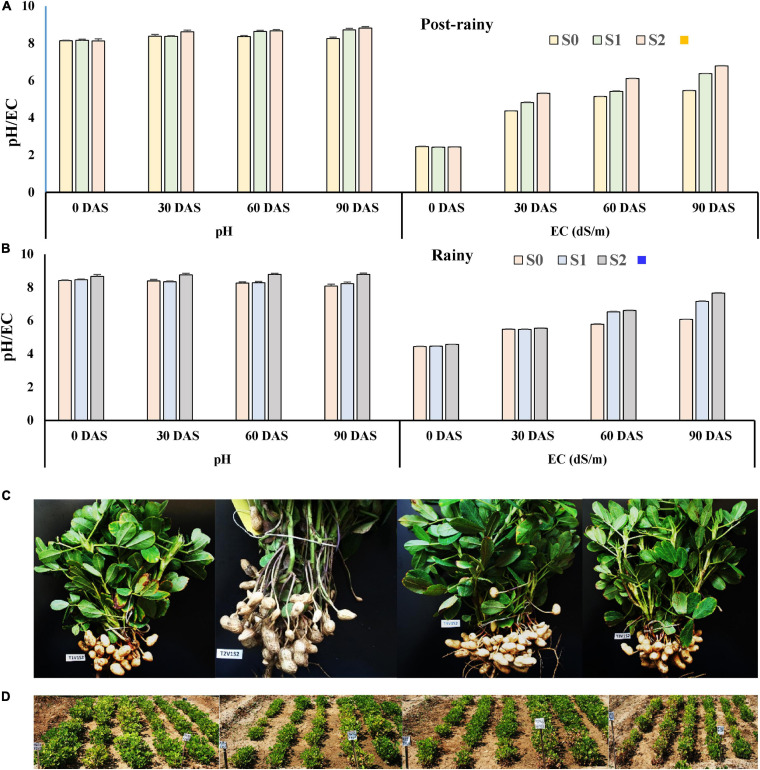
Development of pH and EC in soil due to the application of different levels of saline irrigation water. **(A)** During the post-rainy season of 2013; **(B)** during the rainy season of 2013; data mean of three replications. **(C)** Effect of the application of endophytes on plant growth and pod development: 1st row (L-R): effect on plant growth and pod development of peanut (cv. TG37A) when irrigation water of 6 dS/m is applied, a = uninoculated control, b = inoculated with *Bacillus firmus* J22N, c = inoculated with *Bacillus tequilensis* SEN15N, and d = inoculated with *Bacillus* sp. REN51N; 2nd row: **(D)** field view with application of irrigation water of 3 dS/m: e = inoculated with *Bacillus firmus* J22N (L-R: line 1–3 = GG2, 4–6 = TG37A); f = inoculated with *Bacillus* sp. REN51N (L-R: line 1–3 = TG37A, 4–6 = GG2); 3rd row: field view: g = inoculated with *Bacillus tequilensis* SEN15N (L-R: line 1–3 = TG37A, 4–6 = GG2); h = uninoculated control (L-R: line 1–3 = GG2, 4–6 = TG37A).

The average pH and EC at the beginning of the experiment were 8.14 and 2.44 dS/m (Figure 9A) during the post-rainy season. The pH and EC were monitored at 30 days intervals. The pH of soil did not change much throughout the experiment when saline irrigation water of 1.5–2.0 dS/m was applied. However, application of 3.0 dS/m saline water resulted in a gradual increase in soil pH from 8.16 to 8.37, 8.64, and 8.72 at 30, 60, and 90 DAS, respectively. The EC of the soil also increased from 2.42 dS/m to 4.82, 5.42, and 6.37 dS/m at 30, 60, and 90 DAS (Figure 9A), respectively. Application of 6.0 dS/m of saline water sharply increased the level of salinity of the soil from 2.44 dS/m to 4.82, 5.42, and 6.78 dS/m at 30, 60, and 90 DAS (Figure 9A). The pH also increased from 8.13 to 8.82 during the period of the experiment. As the same plot was used again during the rainy season of 2013 and 2014, the initial pH and EC of soil were at a much higher level. In the plots irrigated with 1.5–2.0 dS/m saline water, the EC increased from 4.44 dS/m at the beginning to 6.07 dS/m at harvest. However, in the case of application of 3.0 dS/m of irrigation water, the salinity of soil increased further from 4.46 dS/m at the time of sowing to 7.16 dS/m at 90 DAS (Figure 9B). However, it ranged from 4.57 dS/m at the beginning to 7.65 dS/m at the harvest (Figure 9B) when irrigation water having 6 dS/m of salinity was used for the experiment. Simultaneously, the pH of the same soil increased from 8.66 at the time of sowing to 8.78 at 90 DAS.

#### Monitoring the Population of Endophytes in the Tissues of Peanut Plants

The population of all the endophytes (root-, stem-, and seed- colonizing) was monitored inside the tissues of the peanut plants at 45 and 90 DAS using the IARs of each isolate (Table 2), both during the post-rainy and rainy seasons. Critical analysis revealed that an increase in salinity has no bearing on the establishment of endophytes inside the peanut tissues; rather with the increase in salinity, the population of endophytes improved inside different tissues across cultivars and season (Figures 6B,C).

As compared to J22N and SEN15N, the root endophyte preferred GG2 as compared to sensitive cultivar TG37A for colonization as evident from the higher population built up in this cultivar across salinity and season. The stem endophyte, SEN15N failed to colonize inside the seed. It was observed that the inoculation of J22N gave population densities of log3.62, log3.68, and log3.67 CFU/g root at 45 DAS at normal saline (S0), 3.0 dS/m (S1), and 6.0 dS/m (S2) level of salinity in TG37A during the rainy season. In a similar situation, it gave log3.73, log 3.74, and log3.67 CFU/g root, respectively at 90 DAS during the post-rainy season. The population of J22N gradually built up in stem and then inside the growing seeds of peanut and gave the population of log4.03, log4.07, and log4.08, respectively at S0, S1, and S2 level of salinity in TG37A at 90 DAS (Figure 6B). It was equally competent at colonizing GG2 as it gave almost the same level of population across three levels of salinity.

As compared to J22N, the stem endophyte SEN15N was established inside the root and stem slowly in both the cultivars, as evidenced by lower the population across different levels of salinity. *Bacillus* sp. REN51N was a more aggressive colonizer in the roots of both the cultivars, preferring GG2 as compared to TG37A, as indicated by the much higher population as compared to the other two endophytes. Across season and cultivars, REN51N was able to colonize all three tissues of peanut, though initially identified as root endophyte (Figures 6B,C). With the increase in salinity, the population of REN51N also increased gradually across cultivars as it gave a population of log3.85, log4.11, and log4.08 CFU/g at 45DAS in the roots of TG37A at S0, S1, and S2 levels of salinity. REN51N also had a population of log4.27, log4.26, and log4.23 CFU/g in GG2 at S0, S1, and S2 level of salinity at 45 DAS (Figure 6B) during the rainy season.

Similar patterns of colonization of the three endophytes were observed in the post-rainy season also with little variation in numbers in different tissues. During the post-rainy season, REN51 was most aggressive in colonizing the root of peanut irrespective of the level of salinity. However, it could establish inside the stem and growing seeds as well. SEN15N, a stem endophyte, failed to colonize seed tissues. Being a seed endophyte, *Bacillus firmus* J22N equally proliferated in root and stem tissues and then started migrating inside the developing seeds, in sizeable numbers, across salinity and cultivars, as evident from its establishment inside the seeds at 90 DAS (Figure 6C). In seeds, J22N gave a population of log3.97, log4.05, and log3.88 CFU/g at S0, S1, and S2 levels of salinity in TG37A at 90DAS. Similarly, in moderately resistant cultivar GG2, it was comparatively more aggressive in colonization and gave log4.03, log4.12, and log4.32 CFU population inside seeds of GG2 at 90 DAS at S0, S1, and S2 levels of salinity (Figure 6C). The root endophyte, besides colonizing root and stem tissues, also colonized seed tissues with a population size of log3.32, log3.79, and log3.69 CFU/g at 90 DAS at S0, S1, and S2 levels of salinity in TG37A, respectively. In contrast, it gave log3.71, log3.65, and log3.61 CFU/g at 90 DAS at S0, S1, and S2 level of salinity in GG2 (Figure 6C).

#### Modulation in the Production of ROS Scavenging Enzymes

**FIGURE 10:**
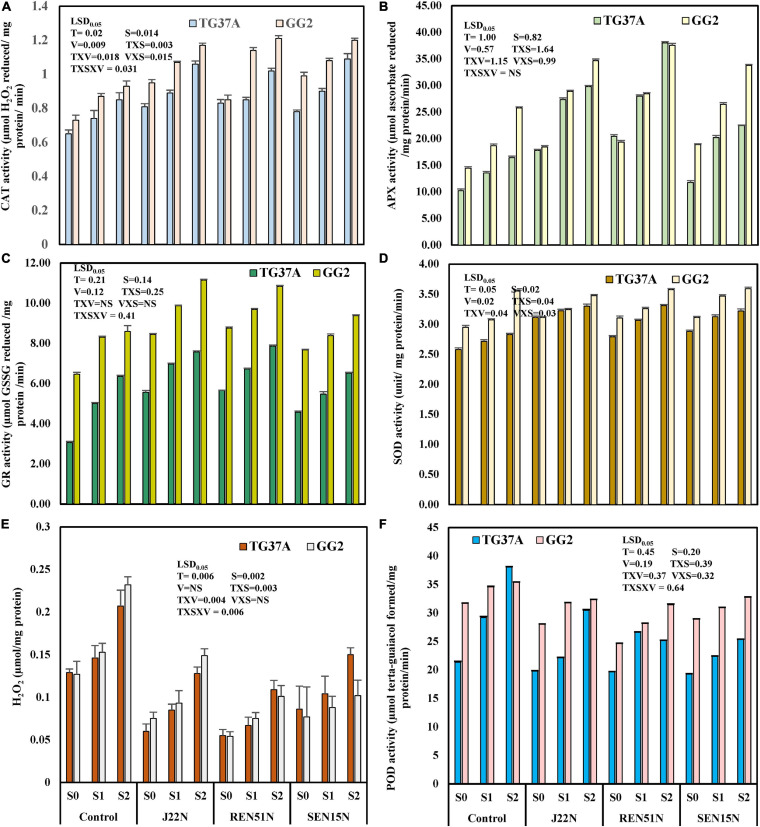
Modulation in production of ROS scavenging enzymes and other biochemical parameters in peanut genotypes (TG37A: susceptible to salinity; GG2: moderately resistant to salinity) by application of endophytic bacteria in field condition at Bhuj during the post-rainy season of 2013. Data mean of three replications, data with the same letter(s) do not differ significantly at *P* = 0.05 according to Tukey’s multiple range test. **(A)** CAT; **(B)** APX; **(C)** GR; **(D)** SOD; **(E)** H_2_O_2_ content; and **(F)** POD activity.

The modulation in the production of enzymes involved in the scavenging of ROS generated inside the leaf of salinity stressed peanut cultivars, and the activities of CAT, SOD, GR, APX, and lipid POD were estimated at 60 DAE in both the post-rainy and rainy seasons. The amount of H_2_O_2_ accumulated as a stressor (due to salinity stress) was also determined. During the post-rainy season, when salinity build-up was comparatively less than in the rainy season, the activities of CAT, APX, GR, and SOD increased significantly with the increase in salinity and increased further with the application of endophytes (REN51N, SEN15N, and J22N) (Figures 10A–D). In addition, the level of activities of these enzymes increased significantly at *P* = 0.05 in GG2 (moderately resistant to salinity) as compared to TG37A (sensitive to salinity). However, the interaction of TxVxS was non-significant at *P* = 0.05 for APX (Figure 10B). Similarly, the activities of POD in the leaf of the peanut plant decreased with the application of endophytes (Figure 10F). The content of H_2_O_2_ increased with salinity significantly but decreased significantly with the inoculation of endophytes (Figure 10E). However, VxS interaction was non-significant at *P* = 0.05 for H_2_O_2_ content. Irrespective of the levels of salinity and treatments, the activities of CAT, SOD, APX, and GR increased significantly by 16.09, 9.47, 19.28, and 51.01%, respectively in GG2 (moderately resistant) cultivar as compared to TG37A (sensitive to salinity). However, the activities of POD decreased significantly by 23.72% in GG2 as compared to TG37A. There was a non-significant difference in H_2_O_2_ content among the cultivars. Similarly, among the endophytes, the inoculation of *Bacillus firmus* J22N significantly enhanced the activities of CAT, SOD, APX, and GR irrespective of levels of salinity and varieties by 24.30, 10.00, 58.27, and 31.19%, respectively (Figures 10A–D) over the control. In identical conditions, the activities of POD and accumulation of H_2_O_2_ in the leaf of peanut plants decreased significantly because of the inoculation of J22N by 13.49 and 40.96%, respectively, over the control. The inoculation of *Bacillus tequilensis* SEN15N resulted in a significant increase of CAT, SOD, APX, and GR irrespective of levels of salinity and variety by 23.37, 7.97, 73.28, and 30.95%, respectively, over control.

**FIGURE 11:**
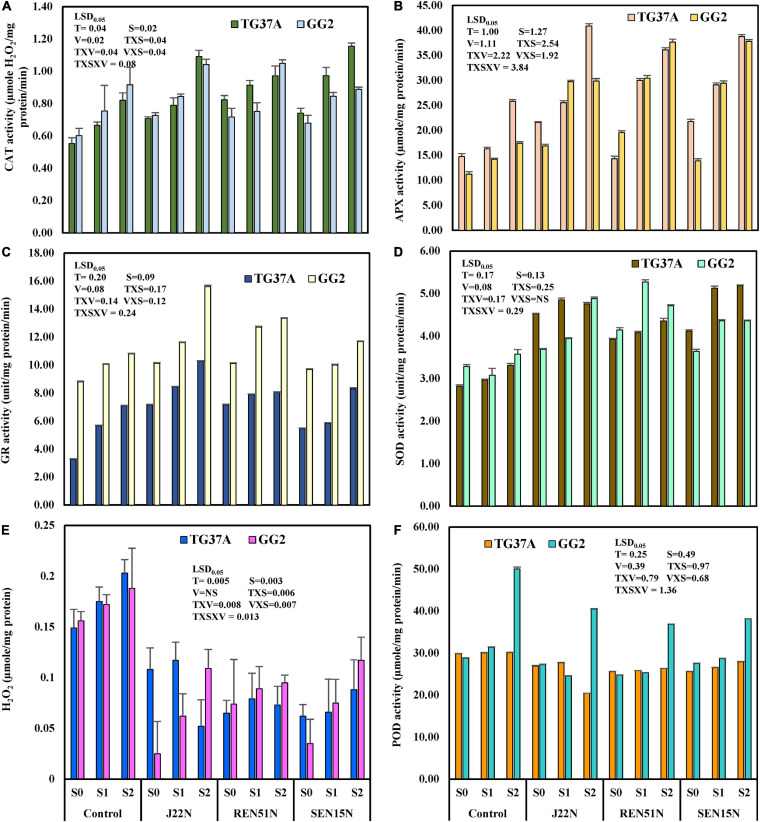
Modulation in production of ROS scavenging enzymes and other biochemical parameters in peanut genotypes (TG37A: susceptible to salinity; GG2: moderately resistant to salinity) by application of endophytic bacteria in field condition at Bhuj during the post-rainy season of 2013. Data mean of three replications, data with the same letter (s) do not differ significantly at *P* = 0.05 according to Tukey’s multiple range test. **(A)** CAT; **(B)** APX; **(C)** GR; **(D)** SOD; **(E)** H_2_O_2_ content; and **(F)** POD activity.

There was a significant decrease in the activity of POD and accumulation of H_2_O_2_ in the leaf of peanut plants by 18.16 and 53.61%, respectively, over the control. However, the application of *Bacillus* sp. REN51N, a root endophyte, significantly improved the activities of CAT, SOD, APX, and GR irrespective of the levels of salinity and variety by 26.34, 9.66, 34.75, and 11.19%, respectively, over the control and decreased the activities of POD by 16.14% and accumulation of H_2_O_2_ by 39.16% over control. During the rainy season when the level of salinity was much more pronounced, the activities of CAT, APX, GR, and SOD increased significantly with an increase in salinity and increased further with the application of endophytes (REN51N, SEN15N, and J22N) (Figures 11A–D). Similarly, the activity of POD and accumulation of H_2_O_2_ increased with salinity but decreased significantly with the application of endophytes (Figures 11E,F). In addition, the interaction of TxVxS was significant at *P* = 0.05 for all the enzymes and accumulation of H_2_O_2_ in the leaf of peanut plants (Figures 11A–F). However, the interaction of SxV was non-significant at *P* = 0.05 for CAT and SOD and the varietal difference in accumulating H_2_O_2_ was non-significant (Figures 11A–F). Irrespective of levels of salinity and treatments, the activities of CAT, SOD, APX, and accumulation of H_2_O_2_ were reduced in resistant cultivar GG2 as compared to sensitive cultivar TG37A, by 3.82, 2.04, 8.50, and 2.91%, respectively. However, there was significantly higher production of GR and POD by 59.28 and 18.71%, respectively in GG2 as compared to TG37A irrespective of the level of salinity and treatments. The inoculation of *Bacillus firmus* J22N significantly enhanced the activities of CAT, SOD, APX, and GR irrespective of levels of salinity and varieties by 20.65, 40.06, 64.92, and 38.46%, respectively (Figures 11A–D) over control. Moreover, the activities of POD and accumulation of H_2_O_2_ in the peanut leaves decreased significantly because of the inoculation of J22N, by 16.43 and 54.60%, respectively, over the control under identical circumstances. The seed bacterization of peanut leaves with *Bacillus tequilensis* SEN15N resulted in a significant increase in CAT, SOD, APX, and GR irrespective of levels of salinity and variety, by 21.26, 39.27, 68.49, and 29.78%, respectively, over the control. There was a significant decrease in the activity of POD and accumulation of H_2_O_2_ in the leaves of the peanut plants, by 17.75 and 54.60%, respectively, over the control. In a similar situation, the application of *Bacillus* sp. REN51N, a root endophyte, significantly improved the activities of CAT, SOD, APX, and GR irrespective of the levels of salinity and variety, by 22.48, 40.85, 71.16, and 11.64%, respectively, over the control and decreased the activities of POD by 12.83% and accumulation of H_2_O_2_ by 57.47.16% over the control. Because of the increase in the level of salinity, the performance of endophytes improved further in modulating the activities of ROS scavenging enzymes.

#### Impact of Endophytes on Pod and Haulm Yield at Elevated Levels of Salinity

The experiment with two varieties and three endophytes was conducted at different levels of application of saline water with S0 (normally available irrigation water salinity of 1.5–2.0 dS/m), S1 (3.0 dS/m), and S2 (6.0 dS/m) levels, for two rainy- (2013 and 2014) and one post-rainy season (2013), in the same plot for successive seasons (Figure 9D). The pod and haulm yield of peanut cultivars, TG3A and GG2, were recorded after harvest.

Analysis of pooled data across the rainy season of 2013 and 2014, irrespective of variety and year, revealed that the interaction was non-significant at *P* = 0.05 for both pod and haulm yield (Table 3). However, treatment was significantly different. It was observed that, irrespective of salinity and variety, the application of *Bacillus firmus* J22N (Figure 9C) improved the pod (1971 kg/ha) and haulm yield (3112 kg/ha) of peanut significantly by 14.74 and 11.25%, respectively (Table 3) over control (pod yield, 1718 kg/ha; haulm yield, 2797 kg/ha). Similarly, the application of root endophyte, *Bacillus* sp. REN51N enhanced the pod (1982 kg/ha) and haulm yield (3263 kg/ha) significantly by 15.36 and 16.68%, respectively over the control (Table 3). The effect of the inoculation of stem endophyte on pod and haulm yield was marginal and improvement was 4.44 and 3.55%, respectively (Table 3).

**TABLE 3:** Field evaluation of endophytes for the alleviation of salinity stress, and enhancement of the growth and yield of peanut genotypes (TG37A: susceptible to salinity and GG2: moderately resistant to salinity) at different levels of salinity (pooled data from the rainy season of 2013 and 2014) across year and genotypes.

Pod yield (kg/ha)						Haulm yield (kg/ha)				
	T1	T2	T3	T4	Mean	T1	T2	T3	T4	Mean
S0	2327	2713	2432	2716	2547	3217	3627	3365	3744	3488
S1	1513	1736	1615	1778	1661	2694	2890	2732	3202	2879
S2	1312	1466	1336	1453	1392	2480	2818	2593	2845	2684
Mean	1718	1971	1794	1982	1866	2797	3112	2896	3263	3017
% Increase		14.74	4.44	15.36			11.25	3.55	16.68	
		SE	CD 5%	CV		SE	CD 5%	CV		
	Y	123.89	NS	13.63		177.64	NS	14.39		
	S	98.1	116.75			76.28	175.68			
	V	42.38	83.91			71.91	142.38			
	T	59.93	118.66			101.70	201.36			
	Interaction	207.6	NS			352.28	NS			

*Y, year; S, level of salinity; S0, irrigation water (1.5–2.0 EC); S1, 3 EC irrigation water salinity; S2, 6 EC irrigation water salinity; T, treatments; T1, control; T2, Bacillus firmus J22N; T3, Bacillus tequilensis SEN15N; T4, Bacillus sp. REN51N; V, variety; V1, TG37A (susceptible to salinity); V2, GG2 (moderately resistant to salinity); 12 irrigations, saline irrigations 10.*

Further analysis revealed that there was an improvement of pod and haulm yield of the varieties by 16.59 and 12.74%, respectively, with the inoculation of *Bacillus firmus* J22N over control, when irrigation water of 1.5–2.0 dS/m (S0) was applied. In similar conditions, the application of *Bacillus* sp. REN51N enhanced the pod and haulm yield by 11.73 and 13.63%, respectively. When irrigation water salinity increased to 3 dS/m (S1), the inoculation of *B. firmus* J22N enhanced the pod and haulm yield of peanuts by 14.74 and 7.28%, respectively. In an identical situation, SEN51N enhanced the pod and haulm yield by 17.51 and 18.86%, respectively, over the control (Table 3). A further increase in irrigation water salinity to 6.0 dS/m resulted in an improvement in the pod yield by 11.74% and haulm yield by 13.63%, over the control, when peanut was inoculated with J22N. Application of REN51N at 6.0 dS/m of irrigation water enhanced the pod yield of peanut by 10.74% and haulm yield by 14.72% over control.

During the post-rainy season of 2013, at the beginning of the experiment, with less build-up of salinity, treatment and interaction were non-significant at *P* = 0.05 (Table 4), with higher pod yield in both the varieties. Though treatment was non-significant, irrespective of variety and levels of salinity, inoculation of *B. firmus* J22N enhanced the pod yield (2329 kg/ha) of peanut by 15.30% over control (pod yield: 2018 kg/ha) (Table 4). It is also evident that the application of *B. tequilensis* SEN15N resulted in enhancement of pod yield (2213 kg/ha against the control of 2018 kg/ha) by 9.55%. The impact of REN51N was more pronounced and enhanced the pod yield (2420 kg/ha) by 19.80% over control. The treatment difference for haulm yield was significant at *P* = 0.05 (Table 4). Application of J22N and REN51N significantly enhanced the haulm yield of peanuts by 12.09 and 16.06% over the control. However, the effect of SEN15N on haulm yield was non-significant.

**TABLE 4:** Effect of application of endophytes on the growth and yield of peanut genotypes at different levels of salinity during the post-rainy season of 2013 with two cultivars (TG37A and GG2) (pooled data across cultivars).

Salinity level	Pod yield (kg/ha)					Haulm yield (kg/ha)				
	T1	T2	T3	T4	Mean	T1	T2	T3	T4	Mean
S0	2232	2642	2496	2826	2549	3249	3457	3408	3802	3479
S1	2059	2279	2254	2415	2252	2747	3179	2802	3043	2943
S2	1763	2066	1889	2020	1934	2265	2622	2302	2740	2482
Mean	2018	2329	2213	2420		2753	3086	2837	3195	
	CD (0.05)					CD (0.05)				
S	341					291				
T	NS					281				
SXT	NS					NS				

*T1, control; T2, Bacillus firmus J22N; T3, Bacillus tequilensis SEN15N; T4, Bacillus sp. REN51N; S0, irrigation water (1.5–2.0 EC); S1, 3 EC irrigation water; S2, 6 EC irrigation water; total irrigation 12; saline irrigation 8.*

## Discussion

Endophytes have co-evolved with plants and help them to acclimatize to the terrestrial ecosystem while transiting from aquatic life. An important bottleneck faced by crop plants has been their inability to maintain growth and biomass production in abiotic stress conditions, resulting in stunted growth and reduced yield. The impairment of growth under salinity stress is due to the effect of salinity on all physiological and biochemical processes, production of an enhanced level of ROS, ion toxicities, disruption of osmotic balance, and water deficits, etc. (Ahmad et al., 2010; Krasensky and Jonak, 2012; Shabala and Munns, 2012; Wu et al., 2013; Acosta-Motos et al., 2017; Kohli et al., 2019). All these issues affect plant growth and need to be addressed to alleviate salinity stress. In this endeavor, endophytes are reported to play a pivotal role in alleviating abiotic stresses. Endophytic modulation in the production of ROS scavenging enzymes, activities of enzymatic antioxidants and lipid peroxidation, signaling pathways, osmotic adjustment, stomatal regulation, and production of antioxidants are a few factors that alleviate stresses. Other factors include the modification in root growth; enhancement of the uptake of essential minerals, particularly K, and the modulation in other metabolic processes (Compant et al., 2005; Ryan et al., 2008; Khaliq et al., 2015; Ali et al., 2017; Li et al., 2019; Fan et al., 2020). Exogenous application of epigallocatechin-3-gallate; salicylic acid; kinetin; silicon; aspirin; Ag-nanoparticles; and spermidine have also been found to alleviate abiotic stresses (Ahammed et al., 2018; Ahanger et al., 2018, 2020; Hussain et al., 2018; Kaur et al., 2018; Zhang et al., 2018; Alam et al., 2019; Ahmad et al., 2019; Khan et al., 2020).

Peanut (*Arachis hypogaea* L.) is one of the important oilseed crops in India. However, its cultivation is badly affected in salinity-affected areas because of excessive use of saline irrigation water, rendering them unfit for cultivation after few years. As peanut cultivars tolerant to salinity are not available at present, alternate ameliorating and alleviating measures are required to bring the salinity-affected areas back to peanut cultivation. To identify microbes capable of such alleviation, 56 root-, stem- and seed- colonizing endophytes were isolated from peanut plants grown in salinity stressed conditions. All the isolates were characterized for the level of tolerance to salinity and production of plant growth promoting traits like ACC deaminase activity and IAA production.

Though all the isolates exhibited tolerance to salinity (5–12.5% of NaCl), thirty-one of them produced ACC deaminase (259–883 ηmol α-ketobutyrate/mg protein/h) and many of them also produced IAA. Thus, endophytes were selected based on ACC deaminase activity as the primary indicator of plat growth promotion as all of them were tolerant to a high level of salinity. Among these, *Pseudomonas pseudoalcaligenes* SEN29N (stem endophyte), *Bacillus* sp. REN51N (root endophyte), and *Bacillus firmus* J22N (seed endophyte) exhibited a high amount of ACC deaminase and IAA production capacity. Thirty-one ACC deaminase-producing endophytes were further evaluated for root growth *in vitro* and alleviation of salinity stress in potted conditions imposing salinity stress. Only nine of the endophytic isolates *Alcaligenes faecalis* J8N, *Pseudomonas otitidis* J11N, *Acinetobacter junii* J20N, *Bacillus firmus* J22N, *Bacillus tequilensis* SEN15N, *Pseudomonas pseudoalcaligenes* SEN29N, *Pseudomonas aeruginosa* REN24N, *Pseudoxanthomonas mexicana* REN47N, and *Bacillus* sp. REN51N produced significantly higher root and shoot length, biomass, and pod yield with salinity sensitive cultivar TG37A at 3.5 dS/m of soil salinity at harvest. There was a significant enhancement in the accumulation of proline with the application of endophytes and reduction in phenol content. This significant increase in plant yield and biomass production could be attributed to the capacity of the endophytes to enhance root and shoot growth by ACC deaminase and IAA production, besides modulating the accumulation of proline as an osmoprotectant.

Production of ACC deaminase, IAA, and accumulation of proline in leaves have been reported to help plants tolerate salinity stress (Glick et al., 2007; Qureshi et al., 2013; Raghuwanshi and Prasad, 2018; Saikia et al., 2018). ACC deaminase activity reduced the ethylene level in roots produced in excess in salinity stress (Trobacher, 2009). Thus, better root growth and biomass production of peanut, cultivar TG37A, might be due to the production of both IAA and ACC deaminase by the endophytes. To evaluate them and understand the mechanisms of alleviation of salinity stress, two sets of field experiments were conducted in field conditions, one with nine endophytes at natural levels of saline water and soil, and another with three endophytes (one each of root-, stem- and seed-endophyte) with three levels of irrigation water salinity on a fixed plot basis.

Among the nine endophytes evaluated, with development of soil salinity (4-6 dS/m of salinity) at harvest, three tissue competent endophytes viz., *Bacillus tequilensis* SEN15N (stem endophyte), *Pseudoxanthomonas mexicana* REN47N (root endophyte), and *Pseudomonas otitidis* J11N (seed endophyte), significantly enhanced biomass in both the seasons along with pod yield during the rainy season. However, pod yield was significantly enhanced by SEN15N only during the post-rainy season. The pod and haulm yield was enhanced in these three endophytes to the tune of 9–23% in pod yield and 7–29% in haulm yield, in identical level soil salinity. Further investigation revealed that inoculation of these endophytes significantly improved relative water content, uptake of potassium, accumulation of proline, and production of ROS scavenging enzymes like CAT, APX, GR, and SOD in both seasons. Furthermore, the accumulation of phenol and H_2_O_2_, and the activity of POD in the leaves of the peanut plants also significantly reduced upon inoculation of these endophytes.

The enzymes, CAT, GR, APX, and SOD, being general scavengers of ROS, reduced the production and accumulation of H_2_O_2_, which is the primary stressor produced in plants in response to salinity stress. These are possible reasons for the alleviation of salinity stress and the enhanced yield of peanuts by inoculation of these three endophytes. Alleviation of stresses and or growth promotion by application of endophytic bacteria have been reported in many plants. This includes *Pseudomonas pseudoalcaligenes* in rice (Jha et al., 2011); *Klebsiella pneumoniae* (Iniguez et al., 2004) and *Enterobacter sakazaki* in soybean (Kuklinsky-Sobral et al., 2004); *Enterobacter* spp. and *Klebsiella* spp. in peanut (Gimenez-Ibanez et al., 2009); and *Bacillus* sp., *Paenibacillus* sp., and *Pantoea dispersa* of peanuts (Li et al., 2019; Fan et al., 2020). Besides these three endophytes (SEN15N, J11N, and REN47N), two other endophytes *Bacillus firmus* J22N and *Bacillus* sp. REN51N also significantly modulated all physiological and biochemical parameters and K-uptake of peanut plants, at the medium level of salinity developed in this experiment, but performance has not been reflected in pod and biomass gain. This might be attributed to the preference of these endophytes to exert the desired effect after certain levels of salinity. However, within the limitation of the work, the reason for such behavior could not be ascertained and needs further investigation.

Few of the isolated endophytes of peanut in the present studies were found to be in the risk group 2 of potential pathogens like *Alcaligenes faecalis*, *Pseudomonas otitidis, Acinetobacter junii*, and *Pseudomonas aeruginosa*: all human pathogens (Clark et al., 2006; Balasubramani et al., 2018; Huang, 2020). However, *Acinetobacter junii* has been reported as a human pathogen rarely. Considering their associated potential hazard, these organisms were not included in the final field trials and hence not recommended for soil application for plant growth promotion and alleviation of salinity stress.

When three endophytes viz. REN51N (root colonizing), J22N (seed colonizing), and SEN15N (stem colonizing) were evaluated further at elevated levels of irrigation water salinity (1.5–2.0; 3.0 and 6.0 dS/m) with two peanut cultivars (TG37A: sensitive; and GG2: moderately resistant), there was a development of soil salinity at harvest between 6.1 and 7.7 dS/m, much higher than the first experiment. All three endophytes were able to colonize the respective niches. Though REN51N was a root endophyte, it was also able to colonize all three tissues along with J22N, but SEN15N did not colonize the seeds. Seed endophytes are important, as they are capable of perpetuating succeeding generations with the seeds. A high level of salinity developed during the rainy season with the application of *Bacillus firmus* J22N and *Bacillus* sp. REN51N significantly improved the pod and haulm yield of peanut plants across cultivars, year, and level of salinity. Whereas the seed inoculation of *Bacillus firmus* J22N improved the pod and haulm yield of peanuts significantly by 14.74 and 11.25%, respectively, *Bacillus* sp. REN51N enhanced the pod and haulm yield significantly by 15.36 and 16.68%, respectively. But inoculation of SEN15N failed to influence pod and haulm yield.

Along with the enhancement of growth and yield of peanut, inoculation of these endophytes significantly enhanced the production of ROS scavenging enzymes like APX, SOD, GR, and CAT and reduced the production of POD and accumulation of H_2_O_2_ in the leaf of peanut. By contrast, in the post-rainy season when salinity level was less (5.5–6.8 dS/m at harvest) than that of the rainy season (6.1–7.7 dS/m), the effect of treatment was non-significant for pod yield. About 10–15% yield enhancement of peanut was obtained with all three endophytes irrespective of variety and level of salinity. Here also, inoculation of endophytes significantly enhanced the production of ROS scavenging enzymes and reduced the accumulation of H_2_O_2_ and activity of POD. At a higher level of salinity stress, SEN15N could not exert its effect on plant biomass and the pod yield of peanuts.

Based on the above, production of ACC deaminase and IAA; modulation of physiological and biochemical parameters; coupled with enhanced uptake of K for counteracting Na^+^ inside plant tissues, underpinned the alleviation of salinity stress and enhanced biomass and peanut yield by inoculation of endophytes. These actions of endophytes have also been reported to alleviate salinity stress in many plants (Zhu, 2002; Sairam and Tyagi, 2004; Compant et al., 2005; Glick et al., 2007; Ryan et al., 2008; Qureshi et al., 2013; Raghuwanshi and Prasad, 2018; Saikia et al., 2018; Fan et al., 2020). *Bacillus firmus* J22N and *Bacillus* sp. REN51N can perform at high levels of salinity (beyond 7.5 dS/m), whereas *Bacillus tequilensis* SEN15N can perform at a medium level of salinity (nearly 6 dS/m).

## Conclusion

In the present study, we identified tissue competent potential root-, stem- and seed- colonizing endophytes of peanut plants for the alleviation of salinity stress in the context of tolerant cultivars not being available due to an increase in soil salinization globally, because of ingression of saline water or increase in the salinity of irrigation water. To address these issues, we identified *Bacillus tequilensis* SEN15N as a potential candidate for the alleviation of salinity stress when cultivating peanuts in soil with a medium level of salinity (4–6 dS/m). *Bacillus firmus* J22N and *Bacillus* sp. REN51N are candidate endophytes for the alleviation of salinity stress in peanut plants at a high level of salinity (6–8 dS/m), for enhancing the growth and yield of peanuts. Enhancement in root and plant growth was due to the production of IAA and ACC-deaminase by the endophytes.

The enhanced uptake of K to counteract Na accumulation inside plant tissues, maintenance of better relative water content vis-a-vis turgidity in leaf, modulation of the production of ROS scavenging enzyme, and enhancement in the accumulation of proline in leaf as osmoprotectant, are involved in nourishing peanut plants at field level soil salinity. Both *Bacillus firmus* J22 and *Bacillus* sp. REN51N can perpetuate successive generations, as they can colonize peanut seeds as they grow. The application of these endophytes helps cultivate peanuts in soil affected by salinity (4–8 dS/m) without much reduction in yield. Moreover, few new genera viz. *Kocuria*, *Brevundimonas*, *Agrococcus*, *Dietzia*, and *Kytococcus* have been found inside the plant tissues of peanuts in this study, which has not been reported elsewhere. As the availability of cultivars tolerant to salinity is still a distant reality, the application of these endophytes as biofertilizers will facilitate sustainable peanut cultivation in salinity-affected areas.

## Data Availability Statement

The raw data supporting the conclusions of this article will be made available by the authors, without undue reservation, to any qualified researcher.

## Author Contributions

RD, KP, Devidayal, SM, and AK designed the experiments along with the analysis of data. DS, RR, MM, PR, RB, MT, MP, PM, BN, SA, and PD conducted the isolation, characterization, and *in vitro* evaluation of all endophytes for physiological and biochemical traits and all other laboratory experiments. KP, RD, and RT wrote the manuscript. All the authors contributed to the article and approved the submitted version.

## Conflict of Interest

The authors declare that the research was conducted in the absence of any commercial or financial relationships that could be construed as a potential conflict of interest.

## Footnotes

1 http://www.ncbi.nlm.nih.gov/
